# PLGF*,* a placental marker of fetal brain defects after in utero alcohol exposure

**DOI:** 10.1186/s40478-017-0444-6

**Published:** 2017-06-06

**Authors:** Matthieu Lecuyer, Annie Laquerrière, Soumeya Bekri, Céline Lesueur, Yasmina Ramdani, Sylvie Jégou, Arnaud Uguen, Pascale Marcorelles, Stéphane Marret, Bruno J. Gonzalez

**Affiliations:** 1 UNIROUEN, Inserm U1245 and Rouen University Hospital, Normandy Centre for Genomic and Personalized Medicine, Normandie University, Rouen, France; 2 Department of Neonatal Paediatrics and Intensive Care, Rouen University Hospital, Rouen, France; 3 Department of Pathology, Rouen University Hospital, Rouen, France; 4 Department of Pathology, Brest University Hospital, Rouen, France; 5 Department of Molecular Biochemistry, Rouen University Hospital, Rouen, France

**Keywords:** Fetal alcohol exposure, Angiogenesis, Cortex, Placenta

## Abstract

Most children with in utero alcohol exposure do not exhibit all features of fetal alcohol syndrome (FAS), and a challenge for clinicians is to make an early diagnosis of fetal alcohol spectrum disorders (FASD) to avoid lost opportunities for care. In brain, correct neurodevelopment requires proper angiogenesis. Since alcohol alters brain angiogenesis and the placenta is a major source of angiogenic factors, we hypothesized that it is involved in alcohol-induced brain vascular defects. In mouse, using in vivo repression and overexpression of PLGF, we investigated the contribution of placenta on fetal brain angiogenesis. In human, we performed a comparative molecular and morphological analysis of brain/placenta angiogenesis in alcohol-exposed fetuses. Results showed that prenatal alcohol exposure impairs placental angiogenesis, reduces PLGF levels and consequently alters fetal brain vasculature. Placental repression of PLGF altered brain VEGF-R1 expression and mimicked alcohol-induced vascular defects in the cortex. Over-expression of placental *PGF* rescued alcohol effects on fetal brain vessels. In human, alcohol exposure disrupted both placental and brain angiogenesis. PLGF expression was strongly decreased and angiogenesis defects observed in the fetal brain markedly correlated with placental vascular impairments. Placental *PGF* disruption impairs brain angiogenesis and likely predicts brain disabilities after in utero alcohol exposure. PLGF assay at birth could contribute to the early diagnosis of FASD.

**Electronic supplementary material:**

The online version of this article (doi:10.1186/s40478-017-0444-6) contains supplementary material, which is available to authorized users.

## Introduction

Fetal alcohol exposure is one of the main causes of mental retardation worldwide and the primary cause of acquired mental retardation in industrialized countries [2]. Fetal alcohol syndrome (FAS), which includes intrauterine growth retardation, characteristic cranio-facial dysmorphism, central nervous system malformations, and neurobehavioral neurocognitive deficits with seizures, is the most severe expression of fetal alcohol spectrum disorders (FASD) [41, 42]*.* Prenatal alcohol exposure causes a continuum of disabilities and most children with in utero exposure do not exhibit the characteristic physical features of FAS [32]. Nevertheless, these infants have neurobehavioral disabilities (attention deficits, hyperactivity), which may remain undetected until they are school age [7]. Diagnosing FASD as early as possible is important for the most appropriate interventions.

Based on research in mice and humans, fetal alcohol exposure affects the brain vasculature by impairing cortical microvessel organization [22]. Preclinical models also showed impacts on the expression of receptors for pro-angiogenic factors belonging to the vascular endothelium growth factor (VEGF) family [22]. In particular, VEGF-R1 is the unique receptor of placental growth factor (PLGF), a member of the VEGF family that is normally weakly expressed in the brain [3], although it can be detected in pathologic conditions [12].

The placenta transfers oxygen and nutrients from the mother to the fetus and removes waste products released by the fetus. With regard to pro-angiogenic factors, trophoblast cells express both VEGF and PLGF [14, 47]. The placenta represents the major source of PLGF during fetal growth [3], and because significant amounts of PLGF appear in the fetal blood [35], it is conceivable that it can reach the fetal brain. Several reports indicate that disruption of VEGF/PLGF balance during pregnancy could be pathogenic [8, 15]. For example, pre-eclampsia features limited endovascular trophoblast invasion and impaired expression of angiogenic factors, which could have a prognostic value in early-onset preeclampsia [15]. Altogether, these data support the hypothesis that altered PLGF expression in the placenta could predict placental vascular pathologies. However, vascular consequences in the fetal brain remain unexplored.

Numerous reports describe the impact of alcohol consumption during pregnancy on placental growth [19, 30]. In addition, up-regulation of VEGF, a permeability inducer and a pro-angiogenic factor, was found in the mouse placenta after acute alcohol exposure [19], and a transcriptomic approach revealed reduced PLGF expression after moderate alcohol exposure [43]. Surprisingly, whereas several animal studies suggest an impact of alcohol on placental angiogenesis [19, 43], characteristic effects of alcohol on the vasculature and the VEGF/PLGF system in human placentae have never been reported.

In summary, alcohol during pregnancy impairs the development of the placenta, which is the main source of PLGF. In addition, high levels of VEGF-R1 are expressed by brain microvessels during development, with angiogenesis in the fetal brain being impaired by alcohol. Hence, we hypothesized that the effects of alcohol on placental pro-angiogenic factors may be associated with vascular defects in the fetal brain. We therefore conducted a preclinical and clinical study to characterize the effects of prenatal alcohol exposure on the brain and placental vasculatures. We also intended to shed light on the effects of prenatal alcohol exposure on the expression of members of the VEGF/PLGF pathway in both the placenta and the brain. Additional goals were to demonstrate that PLGF can reach the fetal brain, to show that PLGF repression in placenta impacts VEGF-R1 expression and vasculature in fetal brains, to determine the impact of placental PLGF overexpression on alcohol-induced vascular defects in the fetal brain and to establish a statistical correlation between placental and brain vascular defects in alcohol-exposed human neonates.

## Materials and methods

### Chemicals

Fast green, Hoechst 33,258, povidone iodine, protease inhibitor cocktail and reagents for electron microscopy were obtained from Sigma Aldrich (Saint-Quentin Fallavier, France). The characteristics of the antibodies raised against CD31, PLGF, VEGFA, VEGF-R1, VEGF-R2, ZO-1, Glut-1, MCT-1 and β-actin (Additional file 1: Table S1). The goat anti-rabbit IgG-HRP (sc-2030) for Western blot experiments, the lentiviral shRNA and the CRISPR-dCas9 plasmids targeting PLGF used for in utero electroporation were obtained from Santa Cruz Biotechnology (Santa Cruz, CA, USA). Alexa Fluor® 488 donkey anti-rabbit IgG (A-21206) and Alexa Fluor® 594 donkey anti-goat IgG (A-11058) used for immunohistochemistry were from Invitrogen. The recombinant human PLGF was obtained from RayBiotech (Norcross, GA, USA) and the human PLGF Elisa kit by Cohesion Biosciences (London, UK). Isoflurane was from Baxter (Maurepas, France). The lysis buffer was from Cell Signaling Technology (Danvers, MA).

### In vivo treatment of pregnant mice

NMRI (National Marine Research Institute) mice (Janvier Labs, Le Genest-Saint-Isle, France) were used according to the recommendations of the French Ethical Committee and the European Directive EC/86/609 (Council Directive 86/609/EEC, license no. 21CAE035), and experiments were carried out under the supervision of authorized investigators (B.J.G., authorization n° 7687 from the Ministère de l’Agriculture et de la Pêche). Modalities of administration, dose of alcohol used for in vivo treatments and the follow-up of blood alcohol levels (BALs) in pregnant mice was defined from a previous study [22]. In particular, injections were performed from GD15 to GD20. Afterwards placentae and brains of the fetuses were collected at GD20 for histological and biochemical studies.

### Visualization and quantification of the cortical microvascular network in GD20 mouse embryos

An immunohistochemical study targeting the endothelial cell marker CD31 was carried out to visualize the brain microvascular network on histological sections from control and alcohol-exposed animals. Immunolabelings were analyzed under a DMI 6000 fluorescence microscope (Leica) equipped with a CCD camera (Roper Scientific, Lisses, France). For vascular network measurements, a morphometric approach was employed using the software Metamorph (Roper Scientific) [22]. In particular, quantification of the angular orientation was performed in the fronto-parietal cortex on two slices per animal and five to seven mice from four different litters per group.

### Immunohistochemistry in mouse brains and placentae

Sections previously fixed with 4% PFA in PBS were incubated overnight at 4 °C with various primary antibodies (CD31, ZO-1, Glut-1, MCT-1, VEGF-R1, VEGF-R2) diluted in incubation buffer (PBS containing 1% bovine serum albumin [BSA] and 3% Triton X-100). Then, the slices were rinsed twice with PBS for 20 min and incubated with the same incubation buffer containing the appropriate secondary antibody. Cell nuclei were visualized by incubating the slices for 5 min with 1 μg/mL Hoechst 33,258 in PBS. Control for nonspecific binding of the secondary antibody was done by omitting the primary antibodies.

### Visualization and histomorphometric quantification of vascular criteria in mouse placenta

Anonymized Cresyl violet stained slices from control and alcohol-exposed placentae were used for blind quantification of the protrusion number and the protrusion length. Practically, z-series of images were acquired and saved in TIFF format with a confocal laser scanning microscope (Leica DMI 6000B microscope and a Leica TCS SP2 AOBS confocal laser scanning imaging system (Leica Microsystems AG). Afterwards, acquired images were deconvoluted using AutoQuant X_3_ software (Media Cybernetics Inc., Rockville, MD, USA) and loaded into IMARIS imaging software (Bitplane, Zurich, Switzerland). Image segmentation was used to discriminate protrusions within the labyrinth zone for 3D reconstruction and quantification of protrusion density and protrusion length. For quantification of the Reichert’s membrane thichness and the density of round shape giant trophoblasts, anonymized toluidin blue stained semithin sections were used. Acquired TIFF format images were opened in the computer-assisted image analysis station Metamorph (Roper Scientific, Evry, France). After calibration of the objective used for acquisitions, thickness and cell density were quantified using the integrated morphometric analysis tools. Because of anatomic specificities between mouse and human placentae (hemotrichorial versus hemomonochorial), morphometric criteria quantified were different between the two species.

### Electron microscopy

Pregnant mice (GD 20) were anesthetized with isoflurane and fixed by intracardiac perfusion of glutaraldehyde 2% in a Sorensen phosphate buffer solution (0.2 M NaH_2_PO_4_, 0.2 M Na_2_HPO_4_, pH 7.3). Placentae were removed and post-fixed 1 h at 4 °C under agitation in the same solution of glutaraldehyde and rinsed at 22 °C in Sorensen phosphate buffer. Tissues were post-fixed with an osmium tetroxide solution 1% and ferricyanide potassium 1.5% in Sorensen phosphate buffer in the dark at 4 °C for 1 h. After three washes of 10 min with Sorensen phosphate buffer, placentae were dehydrated under agitation by successive baths of acetone anhydrous 50–70–90-100%. Samples were embedded in resin epoxy and placed at 60 °C for 48 h for resin polymerization. Semithin and ultrathin sections were cut using an ultra-microtome (Ultracut S, Leica). Semithin sections were stained with toluidine blue and observed under a conventional optic microscope (Leica DMI 6000B) Ultrathin sections were contrasted with uranyl acetate and lead citrate and examined under a Tecnai Biotwin (Hillsboro, OR).

### Visualization and quantification of placenta/fetal brain perfusion by transUV-illumination

To characterize blood perfusion from the placenta to the fetal brain, 3 μL of Evans blue (2% in PBS) were injected into the placenta of GD15 pregnant mice following surgical procedures similar to the in utero transfection protocol. Placentae and their corresponding fetal brains were collected at different times ranging from 10 to 40 min. The fluorescence properties of Evans blue at excitation wavelengths of 530–550 nm were used to visualize and quantify the placenta/brain fluorescence ratio by transUV-illumination using a Bio-Rad Imager (Bio-Rad Laboratories, Marne la Coquette, France).

### Quantification of mouse PLGF levels by ELISA

Placentae from control and alcohol exposed mice were collected at E20 and the labyrinth zone (materno-fetal exchange zone) microdissected. Tissue homogenates were rinced in ice-cold PBS and weighted before homogenization in PBS with a glass homogenizer on ice and ultrasonic cell disruption. ELISA was then performed using the instructions provided in the commercial kit (RayBiotech, Norcross, GA).

### Quantification of human PLGF placenta/fetal brain perfusion by ELISA

Three microliters of human recombinant PLGF (2 ng/μL) were injected in placentae of pregnant mice at GD15 using a surgery protocol similar to the in utero transfection protocol. Thirty minutes after placental injection, whole brains were rapidly collected and a human-specific ELISA kit (Cohesion Biosciences, London, UK) was used to quantify PLGF levels in brain extracts following the instructions provided in the commercial kit.

### In utero placental transfection of lentiviral vectors encoding PLGF shRNA

Pregnant mice timed at GD13 were anesthetized with isoflurane using an anesthetic vaporizer for a maximum of 40 min (Datex-Ohmeda, GE Healthcare, Aulnay sous bois, France). A laparotomy was realized to allow the access to uterine horns. The abdominal cavity, especially the exposed uterine horn, was kept moist with warmed physiological solution. During surgery, the body temperature of the mouse was maintained using a hotplate (Lab-Line Instruments, Melrose Park, IL). Injection of lentiviral plasmids encoding PLGF shRNA was done using micropipettes made of glass capillaries (0.58 mm inner diameter, 1.0 mm outer diameter, Harvard Apparatus, UK) with a P-97 flaming/brown micropipette puller (Sutter Instrument Company; Novato, CA). PLGF shRNA plasmids consisted in a pool of three target-specific lentiviral vectors each encoding 19–25 nt (plus hairpin) shRNAs designed to knock down gene expression (#sc-39,836-SH, Santa Cruz Biotechnology). To follow the injection process, the DNA solution was colored by adding Fast Green solution (1 μg/μL in 0.1 M PBS pH 7.2). The injection depth within the placenta was 0.5 mm and 2 μL of the solution mix injected. For electroporation, the appropriate voltage was applied via specialized platinum electrodes Nepagene CUY 650P (Nepagene Co., Ichikawa, Japan). The voltage conditions were controlled on the NEPA21 type II Electroporator (Nepagene Co., Ichikawa, Japan). After electroporation was done, the uterine horn was carefully replaced in the abdominal cavity and the abdominal walls sutured with sterile Silk Suture Prolene 6–0, MPP2832H (ETHICON, Lidingö, Sweden). Fetal brains corresponding to in utero transfected placentae were collected four days after electroporation for Western blot experiments and vascular morphometric analysis.

### Placental overexpression of PLGF by in utero transfection of *PGF* CRISPR-dCas9 activation plasmids

*PGF* CRISPR-dCas9 activation plasmids (sc-422,211-ACT) constituting the synergistic activation mediator (SAM) complex were designed and provided by Santa Cruz Biotechnology. *PGF* CRISPR-dCas9 activation plasmids were transfected by in utero electroporation at GD13. Surgical procedure was similar to that already described for shRNA plasmid transfection. Alcohol exposure was done from GD15 to GD20 as previously described in the paragraph “*In vivo* treatment of pregnant mice”. The gap of two days between in utero transfection of *PGF* CRISPR-dCas9 activation plasmids and alcohol exposure was required to allow plasmid expression and PLGF over-expression. For a given pregnant mice, 3 placentae were transfected with *PGF* CRISPR-dCas9 activation plasmids, 3 placentae were transfected with negative control CRISPR-Cas9 plasmids (sc-418,922) targeting a non-specific 20 nt guide RNA while other placentae were not transfected and used as internal controls.

### Control and alcohol-exposed human brains

Fetal human cortices were obtained from a collection of archival tissues as previously reported [22]. Sixteen fetal brains ranging from gestational week (GW) 19 to GW38 were subdivided into two groups. Seven brains belonging to the control group were obtained from fetuses whose brains were macroscopically and microscopically free of detectable abnormalities (Additional file 2: Table S2) and whose biometric and maturation data were normal according to Guihard-Costa and Larroche [17] and Fess Higgins and Larroche [11]. Eleven brains were obtained after spontaneous in utero death or after medical termination of the pregnancy for in utero alcohol exposure (Additional file 3: Table S3). For both groups, a complete autopsy had been performed in each case with the informed consent of the parents. Medical termination of the pregnancies had been accepted by the local ethical committee of the Prenatal Diagnosis Multidisciplinary Center according to the French law. Neuropathological data of alcohol-exposed fetuses and neonates are detailed in the Additional file 3: Table S3. In each case, brain growth was evaluated according to the histomorphometric criteria of Guihard-Costa and Larroche [17]. Macroscopic evaluation of brain maturation, in particular gyration, was performed using the atlas of Fess-Higgins and Larroche [11]. Seven-micrometer paraffin-embedded sections were stained using hematoxylin-eosin and cresyl violet, which enabled confirming the absence of cerebral lesions or evaluating the existence of lesions due to prenatal ethanol exposure. The morphology of the brain structures was compared with the age of the patients, which was evaluated by using skeletal measurements, ossification points and the maturational stages of different viscera.

### Control and alcohol-exposed human placentae

Eighty-three placentae from 21 to 42 WG were selected through a collaborative study involving two French centers over a 12-year period (from 2002 to 2013). These placentae were divided in two main groups: a control group (41 placentae) and a group in which maternal alcohol intake sometimes associated with other drug addictions were well documented. Both groups were then subdivided into three subgroups according to the term, i.e. 21 to <25 WG, 25 to <35 WG and 35 to 42 WG. For all subgroups, data from maternal and fetal or neonatal medical history, fetal or neonatal outcome, placental macroscopic and histological examination were provided whenever possible and are summarized in Additional file 4: Table S4 and Additional file 5: Table S5. Fetal biometry was performed according to Guihard-Costa and co-workers [18] and Pinar and co-workers [37].

### Identification of alcohol consuming pregnant women and specific cases

Alcohol consuming pregnant women were identified by obstetricians from the Brest and Rouen University Hospitals after obvious alcohol consumption (drunkenness) either during visit or emergency admission. In some cases, identification of alcohol consumption was associated with blood assays (gamma GT and MGV when available). Two cases presented genetic abnormalities: trisomy 21 (case 1/Additional file 2: Table S2) and trisomy 18 (case 1/Additional file 4: Table S4). Based on a previous report which showed that the separation of maternal serum PLGF levels was small in unaffected and affected (fetal trisomy 18 and trisomy 21) pregnancies, we included these two cases in their respective groups [44]. Concerning twins, only dichorionic diamniotic pregnancies were included.

### Human placental immunohistochemical studies

Six-μm paraffin-embedded sections from central (near cord insertion) were mounted on coated slides (Superfrost Slides, Thermo Scientific, France) and dried overnight in a convection oven (37 °C). Induced epitope retrieval was performed by immersion in a citrate buffer solution pH 6 at 95 °C to 99 °C for 1 h. Incubations were performed for 1 h at room temperature using the Benschmark Ultra system (Ventana Medical Systems, Tucson, AZ), the primary antibody being diluted in an antibody diluant reagent solution (Invitrogen). Primary antibodies used were CD31, VEGF-A, VEGF-R1, VEGF-R2 and PLGF, whose characteristics and working dilutions are described in Additional file 1: Table S1. Peroxidase was visualized using the DAB detection Kit (Ventana Medical Systems). Slides were rinsed in tap water, counterstained with hematoxylin and mounted in mounting medium. Negative controls were obtained by omission of the primary antibody or the use of other antibodies of known reactivity. The distribution of immunoreactive placental components was semiquantitatively evaluated using the following scale: UD, undetected; +, weak immunoreactivity; ++, moderate immunoreactivity; +++, strong immunoreactivity (Additional file 6: Table S6).

### Western blot analysis of cortical and placental extracts from mouse or human

Placentae and/or brain extracts were prepared from control and alcohol-exposed mice and from control and alcohol consuming women. Tissues were homogenized in 300 μL of lysis buffer (Cell Signaling Technology). One hundred micrograms of protein extracts prepared from cortical and placental samples were suspended in Laemmli buffer (100 mM Hepes; pH 6.8; 10% β-mercaptoethanol; 20% SDS), boiled for 5 min, and loaded onto a 10% SDS-polyacrylamide gel. After separation, proteins were electrically transferred onto a nitrocellulose membrane. The membrane was incubated with blocking solution at room temperature for 1 h and incubated overnight with primary antibodies (Additional file 1: Table S1). After incubation with the corresponding secondary antibodies coupled to peroxidase (Santa Cruz Biotechnology, Santa Cruz, CA), proteins were visualized using an enhanced chemiluminescence ECL Plus immunoblotting detection system (Amersham Biosciences Europe GmbH, Freiburg, Germany). The intensity of the immunoreactive bands was quantified using a blot analysis system (Bio-Rad Laboratories, Marne la coquette, France) and β-actin was used as a loading control. Commercial markers (Seeblue prestained standard, Invitrogen) were used as molecular weight standards.

### Visualization and histomorphometric quantification of vascular criteria in human placenta

Vessels were studied by means of CD31 immunohistochemistry. For each placenta, histomorphometric analysis was carried out on sections from the central region using the Metamorph software (Roper Scientific). Analyses consisted of *i)* the quantification and the classification of placental villi according to their size, *ii)* the distribution of vessels per class of villous sizes and the luminal vascular area per class of villous sizes. Analyses were performed on the three groups of ages previously defined. Images (×20 magnification) were acquired using a conventional transmission microscope (Leica DMI 6000B), saved in TIFF format and subsequently opened under the Mercator software (Explora Nova, La Rochelle, France). A grid was affixed on images, allowing for the determination of three regions of interest (ROIs). For each ROI, two parameters were quantified by using the “count objects” application of the software (i.e. number of villi and number of vessels). Moreover, two additional parameters were quantified using the “area” application (i.e. vessel and villous areas).

### Statistical analysis

Statistical analyses were performed using the biostatistic Prism software. Tests used for each experiments, the number of independent experiments and *p* values were summarized in Additional file 7: Table S7.

## Results

### In utero alcohol exposure impairs fetal brain vasculature

**Fig. 1:**
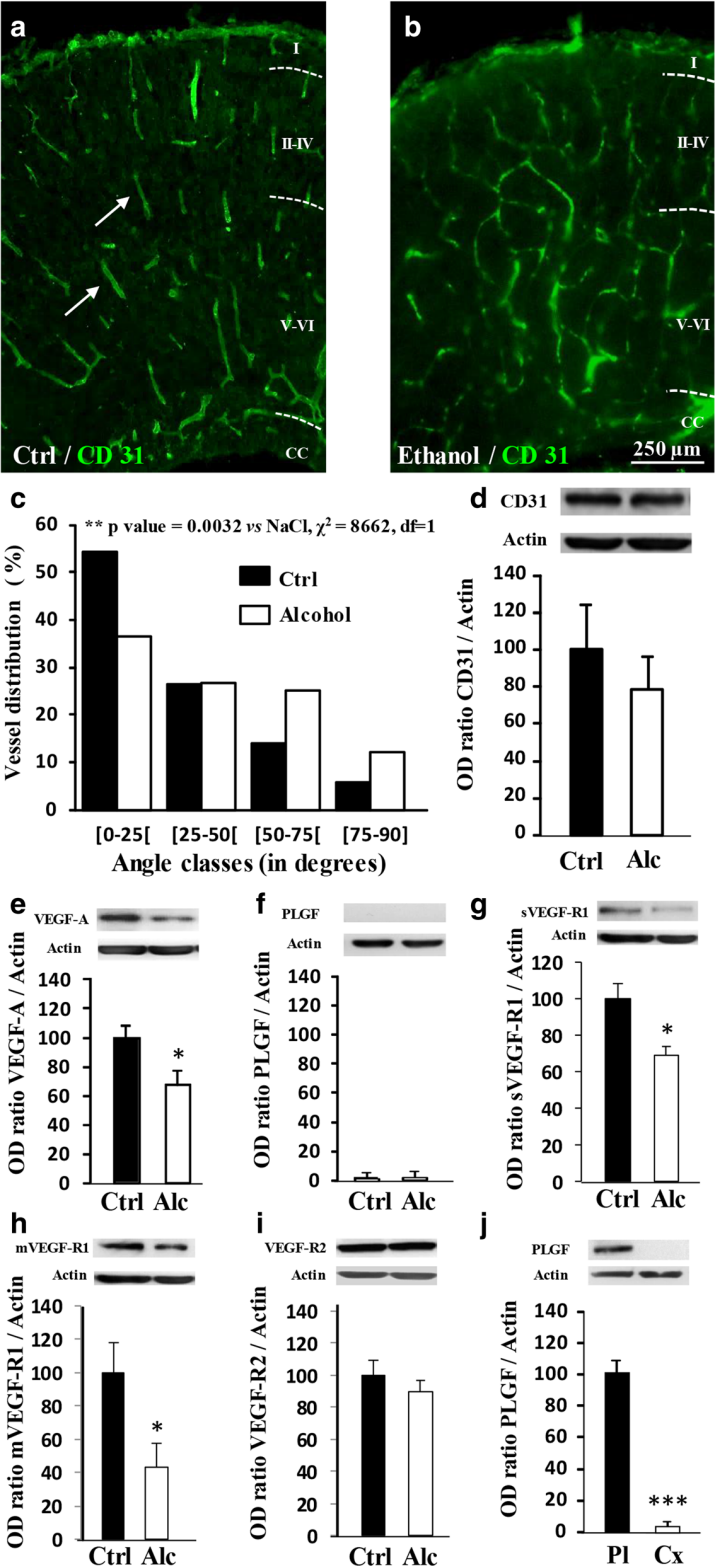
Effects of in utero alcohol exposure on brain angiogenesis and expression of members of the VEGF/PLGF family from E20 embryos. **a**, **b** Effects of fetal alcohol exposure from GD15 to GD20 on the organization of cortical microvessels in control and alcohol-exposed animals. Brain microvessels were visualized by immunohistochemistry against CD31. *Arrows* indicate brain microvessels presenting a radial orientation in the control group. Note a loss of the radial organization in the alcohol-exposed group. I-VI: Cortical layers; CC: Corpus callosum. **c** Distribution of the orientation (angle classes) of cortical microvessels in the immature cortex from GD20 fetuses. Statistical analysis was performed using the χ^2^ test. **d** Quantification by Western blot of the effects of fetal alcohol exposure during the last gestational week on the cortical expression of CD31 at GD20. **e-i** Quantification by Western blot of VEGFA, PLGF, sVEGF-R1, mVEGF-R1 and VEGF-R2 protein levels in the cortex from control and alcohol-exposed groups. *p < 0.05 vs the control group using the unpaired t test. **j** Comparison by Western blot of the PLGF protein levels in the cortex and the placenta of E20 embryos from the control group. ***p < 0.001 vs the control group using the unpaired t test

To model the pathophysiology of FASD in mice, determining if prenatal alcohol exposure alone could affect cortical angiogenesis was necessary. At embryonic day 20 (E20), cortical microvessels from control fetuses were visualized using CD31 immunolabeling and showed a radial organization in all cortical layers during development (Fig. 1a). In contrast, E20 fetuses from pregnant mice that received a daily injection of alcohol (3 g/kg) from GD15 to GD20 had severely disorganized cortical vasculature, particularly in the deepest cortical layers (Fig. 1b). A chi-square statistical analysis indicated that the radial distribution of cortical microvessels was significantly (*p* < 0.01) affected by in utero alcohol exposure, with no reduction in endothelial CD31 expression (Fig. 1c, d). The expression levels of several proteins from the VEGF/PLGF pathway were quantified by Western blot in E20 brain cortices (Fig. 1e-j). In utero alcohol exposure resulted in a 32% ± 9% decrease of VEGF-A (*p* < 0.05; Fig. 1e), whereas PLGF was undetectable by Western blot (Fig. 1f). With regard to VEGF-A and PLGF receptors, both soluble and membrane forms of VEGF-R1 were decreased (*p* < 0.05; Fig. 1g-h), whereas VEGF-R2 had no significant variations (Fig. 1i). To validate the Western blot conditions for PLGF detection, a control experiment compared PLGF protein levels in the fetal cortex at E20 and in the placenta at GD20 (*p* < 0.001; Fig. 1j). These results indicate that alcohol exposure restricted to the fetal life alters cortical angiogenesis in the mouse brain.

### Alcohol exposure impairs the placental integrity and the VEGF/PLGF system

**Fig. 2:**
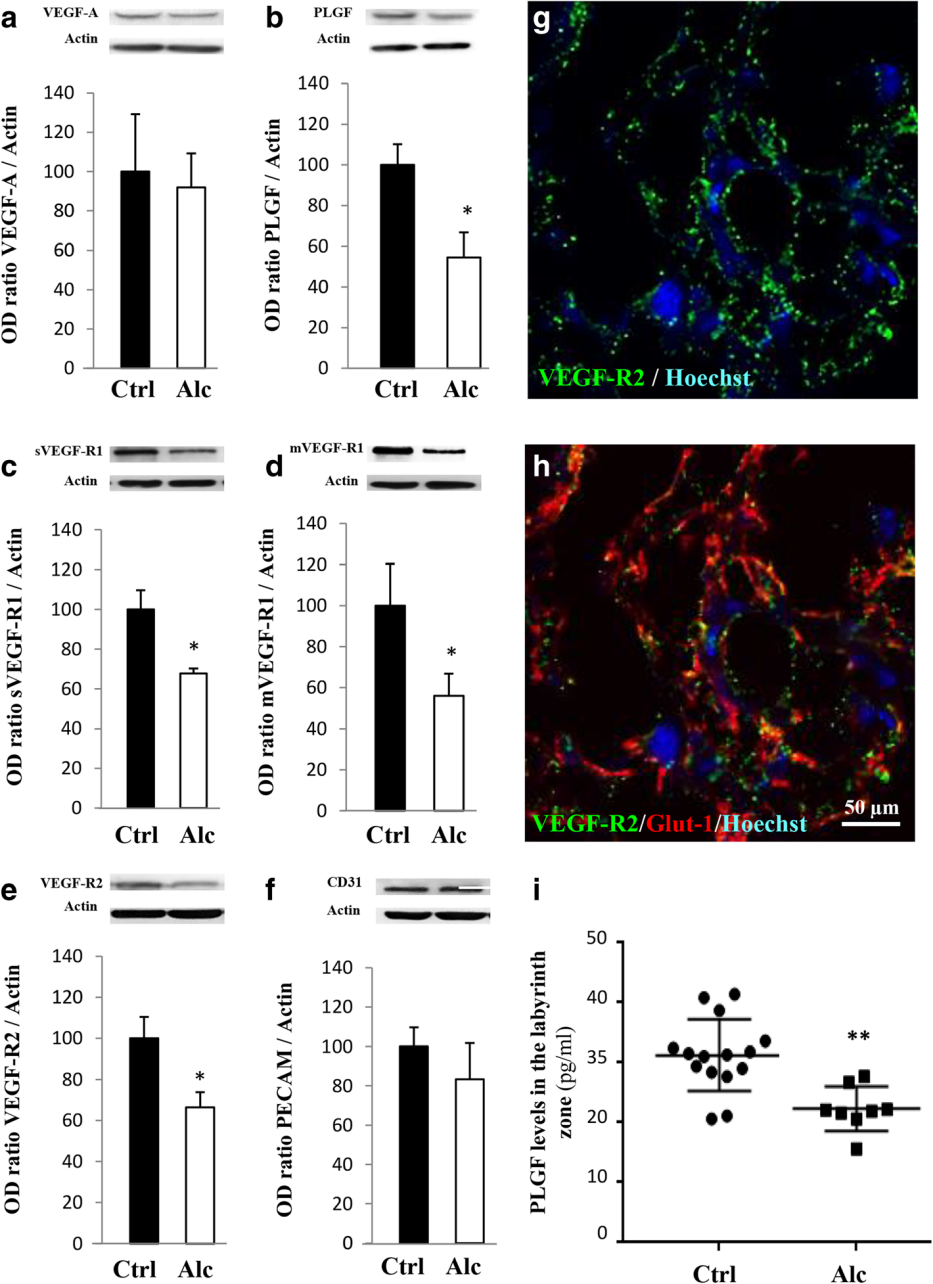
Effects of in utero alcohol exposure on protein expression of members from the VEGF/PLGF family. Quantification by Western blot of the effects of alcohol administered during the last gestational week on the placental expression of VEGF-A (**a**), PLGF (**b**), sVEGF-R1 (**c**), mVEGF-R1 (**d**), VEGF-R2 (**e**) and CD31 (**f**) at GD20. *p < 0.05 vs the control group using the unpaired t test. (**g**, **h**) Immunohistochemistry experiments illustrating the distribution of VEGF-R2 in the syncytiotrophoblast layers of the placenta co-labelled with Glut-1. Hoechst was used to label nuclei. (**i**) Quantification by ELISA of PLGF levels in the microdissected labyrinth zone of control and alcohol-exposed placentae. **p < 0.01 vs the control group using the Mann-Whitney test

Although alcohol has long been known to impair fetal growth [23], studies have only recently focused on metabolic dysfunctions of the placenta [30] and very few reports have targeted the VEGF/PLGF system [19]. In utero alcohol exposure from GD15 to GD20 in mice resulted in abnormal lamination of the placenta with a significant increase of both number and length of protrusions of the junctional zone within the labyrinth zone (*p* < 0.05; *p* < 0.01; Additional file 8: Figure S1a, b, i and j). Reichert’s membrane thickness was significantly reduced (*p* < 0.01; Additional file 8: Figure S1c, d and k), and the morphology of giant trophoblasts was altered (Additional file 8: Figure S1c, d). In the control group, giant trophoblasts possessed a typical rectangular shape (Additional file 8: Figure S1c, l; arrows). In contrast, in the alcohol-exposed group, cell shape was markedly modified and alcohol exposure induced a significant increase of the proportion of “round shape” giant trophoblasts (*p* < 0.0001; Additional file 8: Figure S1d, l; arrows). Electronic microscopy revealed that giant trophoblasts were cohesive in the control group but not in the alcohol-exposed group, in which tight junctions were nearly absent from the placentae (Additional file 8: Figure S1e-h). We also investigated the effect of in utero alcohol exposure on the expression of the tight junction protein ZO-1, the monocarboxylate transporter MCT-1 (Additional file 9: Figure S2a-d), and on placental angiogenic factors from the VEGF/PLGF family (Fig. 2a-f). In alcohol-exposed placentae, PLGF protein expression was reduced (*p* < 0.05; Fig. 2b). Soluble and membrane forms of VEGF-R1 as well as VEGF-R2 protein expression were also significantly reduced (*p* < 0.05; Fig. 2c-e) whereas CD31 expression was not modified (Fig. 2f). VEGF-R1 and VEGF-R2 immunohistochemistry revealed a typical dot-like pattern (Fig. 2g, h and Additional file 9: Figure S2e, f). PLGF levels in the microdissected labyrinth zone was reduced by −28.5% in alcohol-exposed placentae (*p* < 0.01; Fig. 2i and Additional file 9: Figure S2 g). These results indicate that alcohol exposure during pregnancy impairs placenta integrity at the ultrastructural level and the expression of proteins involved in angiogenesis.

### PLGF originating from placenta reaches the fetal brain, affects VEGF-R1 expression and impairs angiogenesis

**Fig. 3:**
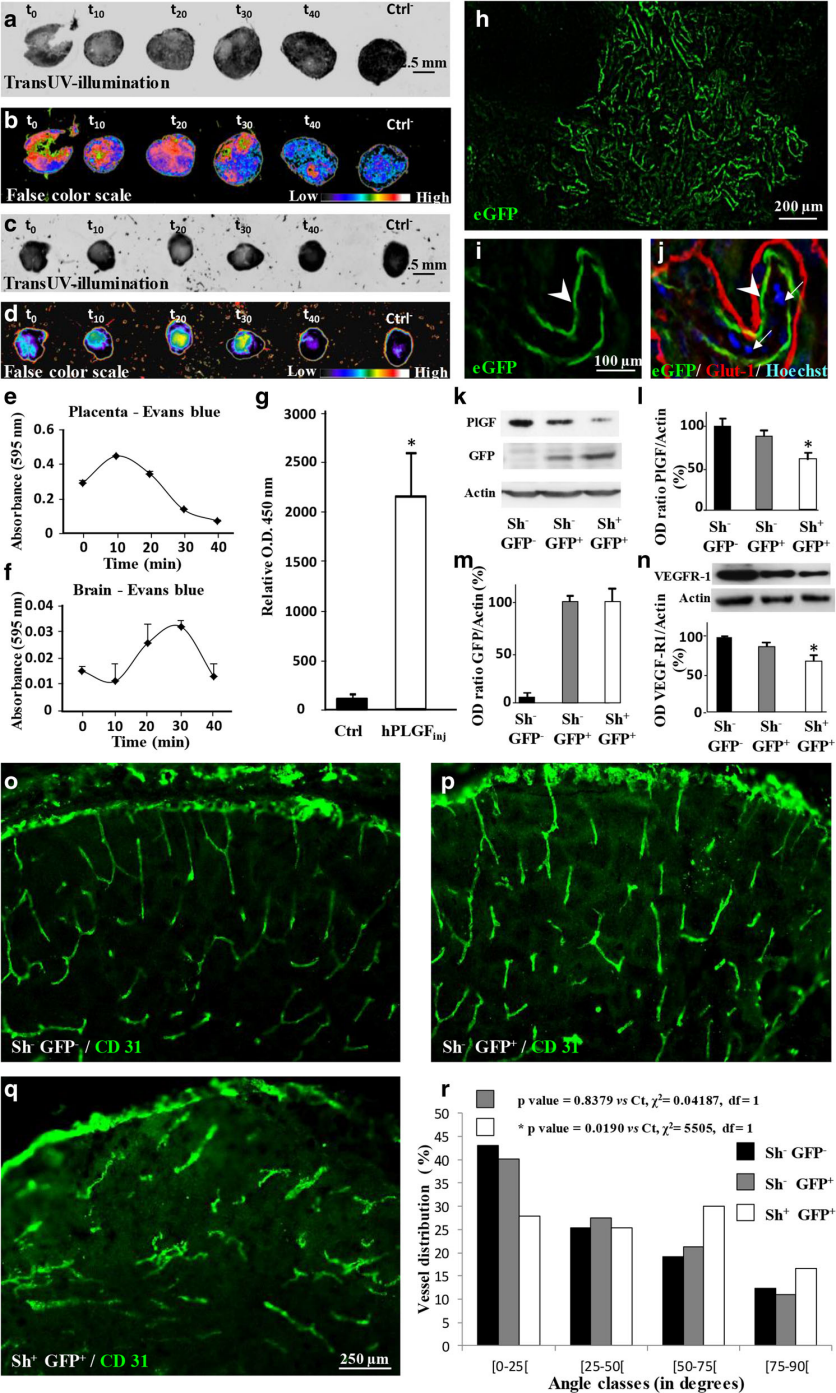
Evans *blue* and hPLGF diffusion from the placenta to the fetal brain and effect of in utero PLGF repression on brain VEGF-R1 levels and cortical vasculature. (**a, b**) Time-course visualization of Evans blue administered by microinjection in the placenta of pregnant mice at GD15. Fluorescence was acquired by transUV illumination (**a**) and visualized with a false color scale (**b**). (**c, d**) Time-course visualization of Evans *blue* fluorescence in the brain of fetuses after a placental microinjection at GD15. Fluorescence was acquired by transUV illumination (**c**) and visualized with a false color scale (**d**). (**e, f**) Time-course quantification by spectrophotometry of the 595 nm absorbance of the Evans *blue* signal injected in placentae (**e**) and the follow-up in the corresponding fetal brains (**f**). (**g**) Quantification by ELISA of human PLGF in the brain of fetuses 30 min after injection in the placentas of pregnant mice at GD15. *p < 0.05 vs the control group using the unpaired t test. (**h**) Microphotograph visualizing eGFP expression 48 h after in utero transfection of placentae from GD15 pregnant mice with an eGFP encoding plasmid. (**i, j**) triple staining eGFP/Glut-1/Hoechst indicating that eGFP fluorescence (**i**) is mainly associated with the fetal trophoblastic layer (**j**) labelled with Glut-1 (*arrow heads*). Note that the maternal trophoblastic layer which is also labelled by Glut-1 is poorly transfected. The fetal side of the trophoblastic layers is identified by the presence of nucleated *red* blood cells characteristic of the fetal circulation (*arrows*). (**k**) Visualization by Western blot of PlGF, GFP and actin proteins in placentae from non-transfected (sh^−^/GFP^−^), GFP-transfected (sh^−^/GFP^+^) and shPLGF/GFP transfected (sh^+^/GFP^+^) animals. (**l**, **m**) Quantification by Western blot of PLGF and GFP expression levels in placentae from non-transfected (sh^−^/GFP^−^), GFP-transfected (sh^−^/GFP^+^) and shPLGF/GFP transfected (sh^+^/GFP^+^) animals four days post-transfection. (**n**) Quantification by Western blot of VEGF-R1 expression levels in the brain of fetuses from non-transfected (sh^−^/GFP^−^), GFP-transfected (sh^−^/GFP^+^) and shPLGF/GFP-transfected (sh^+^/GFP^+^) placentae four days post-transfection. *p < 0.05 vs the “sh^−^/GFP^−^” group using the one way ANOVA test followed by Tukey’s post hoc test. (**o-r**) Visualization of the vasculature in the cortex of fetuses from non-transfected (sh^−^/GFP^−^) (**o**), GFP-transfected (sh^−^/GFP^+^) (**p**) and shPLGF/GFP-transfected (sh^+^/GFP^+^) (**q**) placentae. Statistical analysis of vessel disorganization was performed using the χ^2^ test (**r**)

Whereas VEGF-R1 is expressed in the fetal brain (Fig. 1g, h), PLGF is massively expressed by the placenta (Fig. 1f, j) [3], suggesting that some alcohol-induced brain vascular defects may result from placental angiogenic factors. To confirm this hypothesis, we performed transUV-illumination experiments after in utero placental injections in mice (Fig. 3a-d). In time-course studies, Evans blue fluorescence was immediately detectable in the placenta after in utero injection (Fig. 3b, e). Fluorescence reached a maximum at 10 min and then progressively decreased (Fig. 3e). Evans blue fluorescence was also detected in the matched fetal brains 20 to 30 min after placental injection (Fig. 3d, f). Through the same protocol, human recombinant PLGF was injected into the placenta of pregnant mice at GD15. A specific hPLGF ELISA detected recombinant hPLGF in the fetal brain 30 min after the injection (*p* < 0.05; Fig. 3g). Moreover, PLGF was detected by Western blot in the cephalic blood of E20 fetuses (Additional file 9: Figure S2 h). Altogether these data indicate that pro-angiogenic factors released by the placenta can reach the fetal brain.

Daily injection of pregnant mice with alcohol from GD15 to GD20 resulted in decreased VEGF-R1 protein levels in the fetal brain (Fig. 1g, h). To determine if PLGF is involved in this effect, a shRNA strategy coupled with in utero placenta transfection was conducted (Fig. 3h-n). Electroporation of an eGFP-expressing vector revealed that the syncytiotrophoblast layer cells expressed eGFP 48 h post transfection (Fig. 3h). Triple fluorescent labeling indicated that fetal syncytiotrophoblasts were efficiently transfected (Fig. 3i, j; arrow heads). The presence of nucleated red blood cells identified the fetal syncytiotrophoblast layer (Fig. 3j; arrows) [46]. In non-transfected placentae (Sh^−^/GFP^−^), PLGF was detected by Western blot, and no eGFP signal was found (Fig. 3k–m). In the Sh^−^/GFP^+^ condition, eGFP was detected in placental extracts, while PLGF levels were not significantly affected (Fig. 3k–m). In placentae transfected with a plasmid encoding PLGF shRNA (Sh^+^/GFP^+^), PLGF protein levels were significantly reduced by 38% ± 5% (*p* < 0.05; Fig. 3l). In the Sh^−^/GFP^+^ condition, cortical protein levels of VEGF-R1 decreased slightly but not significantly after placental electroporation, whereas PLGF repression (Sh^+^/GFP^+^) resulted in a significant decrease of cortical VEGF-R1 levels (*p* < 0.05; Fig. 3n). Four days after in utero transfection of placentae with Sh^+^/GFP^+^ plasmids (Fig. 3o-r), PLGF repression induced a marked impairment of the vasculature in the fetal brain (Fig. 3q, r). No effect on the brain vasculature was found in the Sh^−^/GFP^+^ group (Fig. 3p). These data indicate that repression of placental PLGF alters brain VEGF-R1 expression and impairs cortical angiogenesis of the fetus, supporting the idea of placental contribution to alcohol-induced brain vascular defects.

### Overexpression of placental *PGF* rescues alcohol-induced angiogenesis defects in the fetal brain

**Fig. 4:**
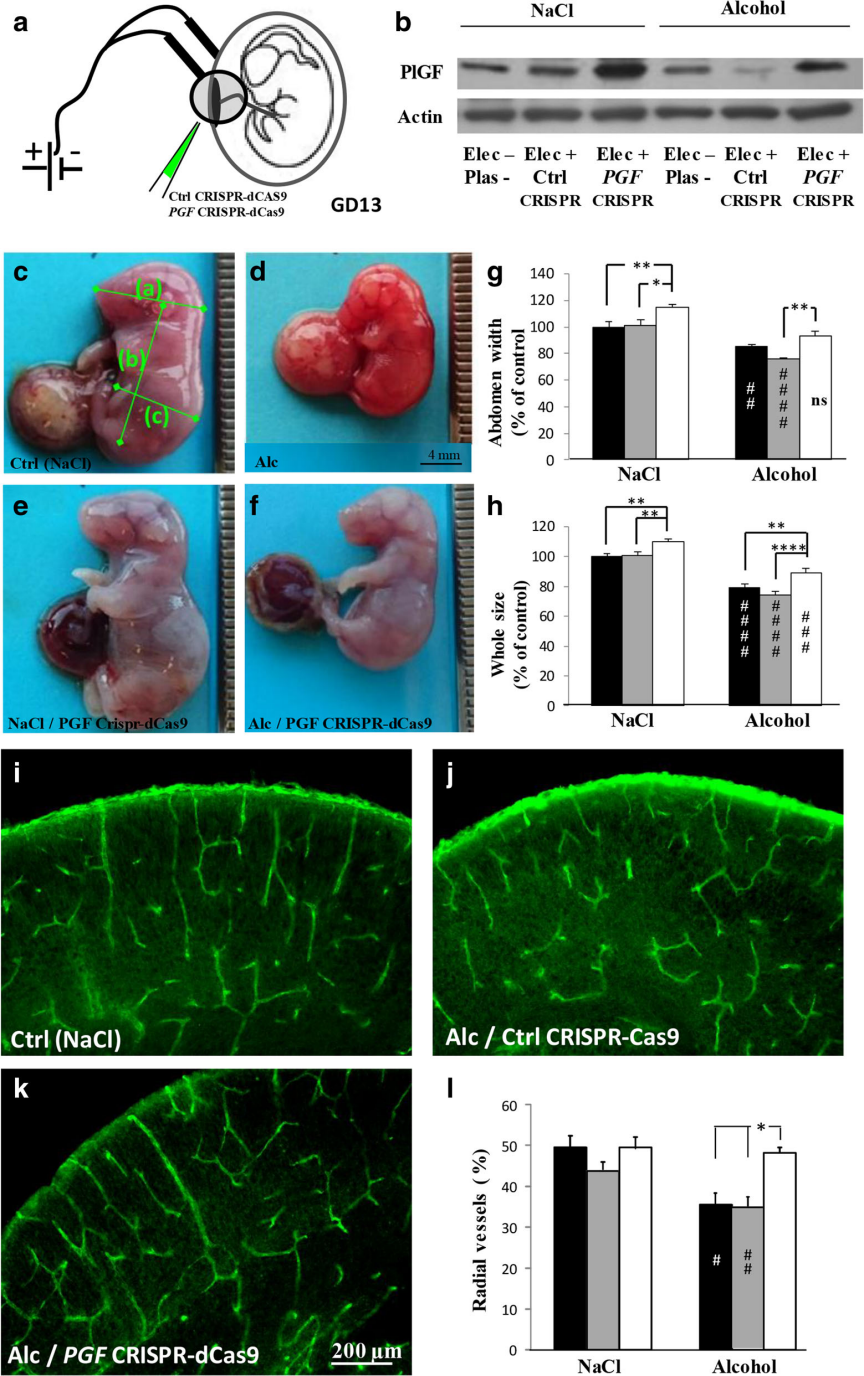
Effects of in utero *PGF* overexpression on fetal growth and cortical vasculature during prenatal alcohol exposure. **(a**, **b)** *PGF* CRISPR-dCas 9 activation approach coupled with in utero electroporation of the placenta was done at GD13 (**a**) and overexpression of PLGF controlled at GD20 (**b**). In the alcohol group, in utero exposure occurred from GD15 to GD20. **(c**, **d)** Visualization of E20 fetuses from pregnant mice exposed to NaCl (**c**) or alcohol (**d**). Note the small size of alcohol-exposed fetuses. *Green bars* indicate morphometric measures that have been done (head size (a); body size (b); abdomen size (c) and whole fetus size (a + b). **(e, f)** Visualization of E20 fetuses after in utero electroporation of *PGF* CRISPR-dCas9 plasmids in placentae from control (**e**) or alcohol-exposed pregnant mice (**f**). **(g, h)** Quantification of abdomen (**g**) and whole fetus (**h**) sizes in control (NaCl) and alcohol groups. In a same uterine horn some placentae were not electroporated (*black bars*), electroporated with control CRISPR-Cas9 plasmids (grey bars) or electroporated with *PGF* CRISPR-dCas9 plasmids (*white bars*). ##*p* < 0.01; ###*p* < 0.001; ####*p* < 0.0001 vs the control group and *p < 0.05; **p < 0.01; ****p < 0.0001 as indicated using the two way ANOVA test followed by Tukey’s post hoc test. **(i-k)** Visualization of the vasculature in the cortex of E20 fetuses from control (NaCl)/non-transfected (**i**), alcohol/control CRISPR-Cas9 transfected (**j**) and alcohol/ *PGF* CRISPR-dCas9 transfected (**k**) placentae. **(l)** Quantification of the percentage of radial vessels in the cortex of E20 fetuses from not electroporated (*black bars*), electroporated with control CRISPR-Cas9 plasmids (*grey bars*) and electroporated with *PGF* CRISPR-dCas9 plasmids (*white bars*) placentae. #*p* < 0.05 vs the control group and *p < 0.05 as indicated using the two way ANOVA test followed by Tukey’s post hoc test

The CRISPR-dCas9 activation strategy coupled with in utero placenta transfection was used to allow for robust induction of endogenous *PGF* gene expression in the placenta of control and alcohol-treated pregnant mice (Fig. 4a, b). At GD20, in utero alcohol exposure resulted in intrauterine growth restriction of fetuses (Fig. 4c, d) with a significant reduction of the head (*p* < 0.01; Additional file 10: Figure S3a), body (*p* < 0.0001; Additional file 10: Figure S3b), abdomen (*p* < 0.01; Fig. 4g) and whole fetus (*p* < 0.0001; Fig. 4h) sizes. In the control group, *PGF* overexpression induced macromorphic fetuses (Fig. 4c, e) with a significant increase of the abdomen (*p* < 0.01; Fig. 4g) and whole fetus (*p* < 0.01; Fig. 4h) sizes. In the alcohol group, *PGF* overexpression significantly increased body (*p* < 0.05; Additional file 10: Fig. S3b) and whole fetus (*p* < 0.01; Fig. 4h) sizes. When compared to the control group, *PGF* overexpression abolished the effects of alcohol on head (Additional file 10: Figure S3a) and abdomen (Fig. 4g) sizes while it reduced by 38.6 ± 2.8% and 46.8 ± 2.9% the effects of alcohol on body (Additional file 10: Figure S3b) and whole fetus sizes (Fig. 4h), respectively. No effect on fetus morphology was found in pregnant mice transfected with control CRISPR-Cas9 plasmids (Fig. 4g, h). In the brain of E20 fetuses, prenatal alcohol exposure resulted in a disorganization of the cortical vasculature (Fig. 1a, b). Transfection of placentae with control CRISPR-Cas9 plasmids had no effect on the angiogenic defects induced by in utero alcohol exposure (Fig. 4i–l). In contrast, in pregnant mice transfected with *PGF* CRISPR-dCas9 activation plasmids, the radial organization of the cortical microvessels was significantly restored (*p* < 0.05; Fig. 4k, l). These data constitutes the first demonstration that PLGF overexpression in the placenta is able to rescue, almost in part, morphometric and vascular impairments induced by in utero alcohol exposure.

### In utero alcohol exposure induces histomorphometric defects in human placenta

**Fig. 5:**
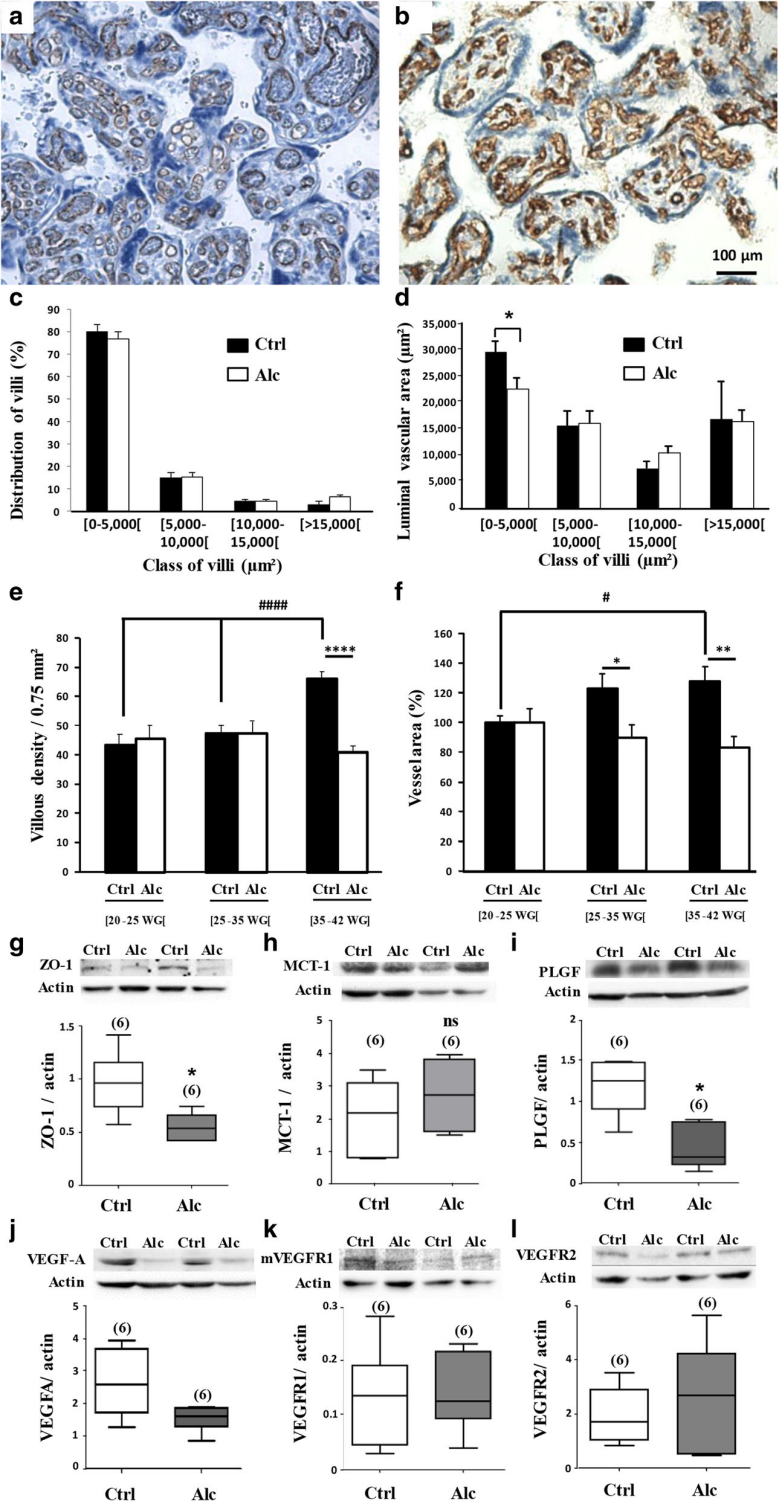
Effects of in utero alcohol exposure on histomorphometric characteristics of human placentae and on the expression of proteins from the placental barrier, the energy metabolism and the VEGF/PLGF family. **a, b** Immunohistochemistry performed against CD31 and toluidine *blue* counterstaining visualizing microvessels (*brown*) present in placental villi (*blue*) from control and alcohol-exposed groups collected at gestational ages ranging from [35–42 WG]. Note the marked reduction of the luminal area of microvessels in the alcohol-exposed group. **c** Percentage of villi classified by sizes in placentae from control and alcohol-exposed groups collected at gestational ages ranging from [35–42 WG]. **d** Luminal vascular area per size of villi in placentae from control and alcohol-exposed groups collected at gestational ages ranging from [35–42 WG].*p < 0.05 vs the control group using the unpaired t test. **e** Time-course of the villous densities in placentae from control and alcohol-exposed groups for classes of gestational ages [20–25 WG], [25–35 WG] and [35–42 WG]. ^####^ *p* < 0.0001 vs Ctrl [20–25 WG] after one way ANOVA analysis; ****p < 0.0001 between Control and Alcohol groups after impaired t test analysis. **f** Time-course of the vessel area in placentae from control and alcohol-exposed groups for classes of gestational ages [20–25 WG], [25–35 WG] and [35–42 WG].^#^ *p* < 0.05 vs Ctrl [20–25 WG [after one way ANOVA analysis; *p < 0.05 between Control and Alcohol groups after impaired t test analysis. **g-l** Quantification by Western blot of ZO-1, MCT-1, PLGF, VEGFA, VEGF-R1 and VEGF-R2 protein levels in human placentae from control and alcohol-exposed groups. *p < 0.05 vs the control group using Mann and Whitney test

Placentae from control (Additional file 4: Table S4) and alcohol-consuming (Additional file 5: Table S5) pregnant women were classified into three groups: 20 to <25 weeks of gestation (WG), 25 to <35 WG and 35 to 42 WG. In the 20 to <25 WG group, numerous small villi containing small vessels were observed (Additional file 11: Figure S4). The proportion of small villi was significantly lower in the alcohol-exposed group (*p* < 0.05; Additional file 11: Figure S4c), but no other major differences were found between the two groups (Additional file 11: Figure S4a-e). The 25 to <35 WG placentae had a marked morphologic difference between the control and alcohol-exposed groups (Additional file 12: Figure S5). In controls, villi contained vessels with large luminal sections, but in placentae from the alcohol-exposed group, the vessels had small lumens and the lumen area in small size villi was significantly lower (*p* < 0.05; Additional file 12: Figure S5a, b and e). The diameter of vessels was also reduced in the 35 to 42 WG alcohol-exposed group compared to the control group (Fig. 5a, b) indicating that a significant reduction of the luminal vascular area persisted until birth (*p* < 0.05; Fig. 5d).

We also performed a time-course study of villous and vessel densities (Fig. 5e, f). In the control group, villous density was similar between 20 to 35 WG (Fig. 5e) then increased from 35 to 42 WG (*p* < 0.0001; Fig. 5e). In the alcohol-exposed group, villous density was similar to the control group from 20 to 35 WG (Fig. 5e), but the massive increase of the villous density observed in the control group during the last 2 month of gestation did not occur in the alcohol-exposed group (*p* < 0.0001; Fig. 5e). Moreover, in the control group, the vessel intravillous area regularly increased during the gestation (*p* < 0.05; Fig. 5f) in contrast to the alcohol-exposed group, in which vessel area tended to decrease (*p* < 0.05; Fig. 5f). These data constitute the first demonstration that alcohol has deleterious effects on human placental vasculature.

### In utero alcohol exposure impairs the VEGF/PLGF system in human placenta

Since histomorphometric data showed major differences from 35 to 42 WG (Fig. 5a–f), further analyses were performed at these stages by Western blot. A significant decrease in the expression of ZO-1 was found in placentae from women who consumed alcohol (*p* < 0.05; Fig. 5g). Levels of MCT-1 tended to increase, but statistical analysis showed no significance (Fig. 5h). PLGF and VEGF-A levels were also quantified, and significant decreases were found regarding PLGF in the alcohol-exposed group (*p* < 0.05; Fig. 5i, j). Quantification of VEGF-R1 and VEGF-R2 indicated no differences between control and alcohol-exposed groups (Fig. 5k, l). We then performed immunohistochemical studies using VEGF, PLGF, VEGF-R1 and VEGF-R2 antibodies in both control and alcohol-exposed human placentae (Additional file 6: Table S6 and Additional file 13: Figure S6). The two groups had some differences in PLGF immunoreactivity, which was lower in the alcohol-exposed group than in the control group in extravillous and intravillous trophoblasts, as well as in decidual and intravillous vessel endothelial cells. In particular, after 34 WG, the alcohol-exposed group showed very low immunoreactivity, whereas it was apparent in villous vessel lumens of controls (Additional file 6: Table S6 and Additional file 13: Figure S6). VEGF-R1 and R2 immunoreactivity did not differ among groups and cell types except for villous trophoblasts and endothelial cells in which VEGF-R1 and R2 immunoreactivity was low from 35 WG in the alcohol group (Additional file 6: Table S6). These data indicate that in utero alcohol exposure induced major differences in the expression profile of proteins involved in angiogenesis in human placenta during the third trimester of pregnancy.

### Placental impairments correlate with brain vascular defects in human

**Fig. 6:**
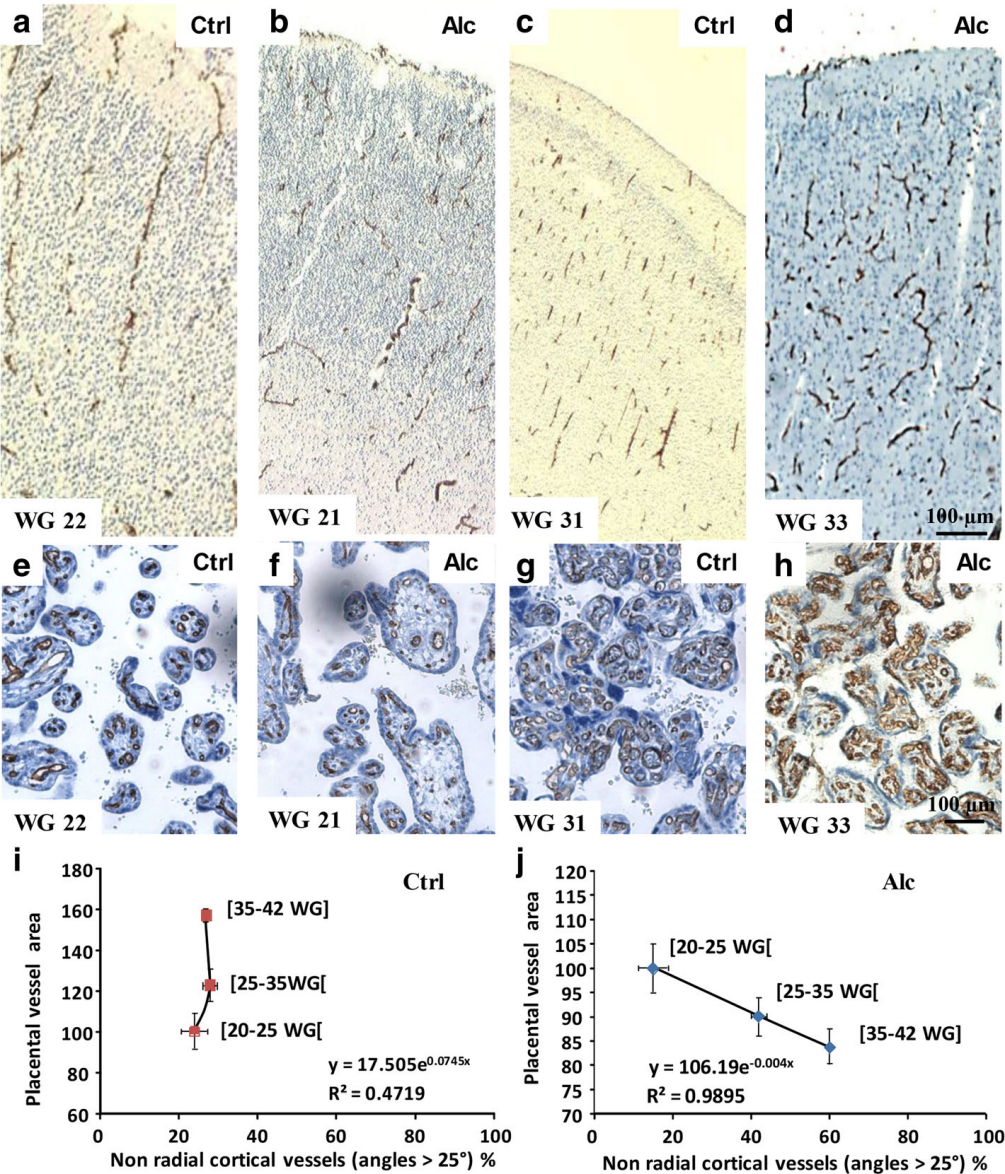
Brain/placenta comparisons of in utero alcohol-induced defects in human. (**a-h**) Comparative visualization of the vascular organization in the brains (**a-d**) and the placentae (**e**-**h**) of control and alcohol-exposed groups. Two developmental windows are shown: [21–22] WG and [10, 32, 33] WG. Statistical correlation between cortical and placental vascular impairments in patients from the control (**i**) and the alcohol-exposed groups (**j**)

Since preclinical data showed that prenatal alcohol exposure induced disorganized orientation of cortical microvessels (Fig. 1), impaired expression of placental angiogenic factors (Fig. 2), that targeted repression of PLGF in the placenta mimicked the effects of in utero alcohol exposure on both VEGF-R1 expression and vessel organization in the fetal brain (Fig. 3) and that placental over-expression of PLGF rescued alcohol-induced vascular defects (Fig. 4), we researched in human an association between vascular defects found in the placentae and in the brain after in utero alcohol exposure. Immunohistological studies showed that the cortical orientation of brain microvessels was similar between control and alcohol-exposed groups from 20 to 25 WG (Fig. 6a, b), with most vessels being radially oriented. In addition, placental vasculature did not differ (Fig. 6e, f). In contrast, from 35 to 42 WG, cortical microvessel organization was markedly impaired in the alcohol-exposed group (Fig. 6c, d) along with vessel luminal area in the placentae (Fig. 6g, h). In the control group, no correlation existed between cortical vessel organization and placental vessel area; the radial organization of cortical microvessels remained unchanged between gestational groups, while placental vessel area strongly increased during gestation (Fig. 6i). In contrast, in the alcohol-exposed group, the disorganized orientation of the cortical microvessels massively increased with pregnancy duration (Fig. 6j) and was markedly correlated with the lack of increase of placental vessel area (Fig. 6j).

## Discussion

Using preclinical and clinical approaches, we investigated the effects of prenatal alcohol exposure on both brain and placental vasculatures. We demonstrated that the angiogenesis and the expression of VEGF/PLGF proteins are altered in both placentae and fetal brains. We also showed that PLGF can reach the fetal brain and that targeted in utero repression of PLGF in the mouse placenta mimics the effect of prenatal alcohol exposure on both VEGF-R1 expression and vasculature impairments in the fetal brain. In addition, PLGF overexpression by *PGF* CRISPR-dCas9 activation rescues brain vascular defects induced by in utero alcohol exposure. Our results in mice are similar to those observed in humans, with placental and brain vascular defects being strongly correlated in alcohol-exposed human fetuses. Since decreased PLGF levels in the placenta after in utero alcohol exposure are associated to brain angiogenesis defects, the levels may serve as a predictive marker for subsequent neurodevelopmental outcomes of exposed fetuses. Compared with the known exposition markers of maternal alcohol intake, this new generation of “effect” biomarkers could facilitate early diagnosis of FASD.

**Fig. 7:**
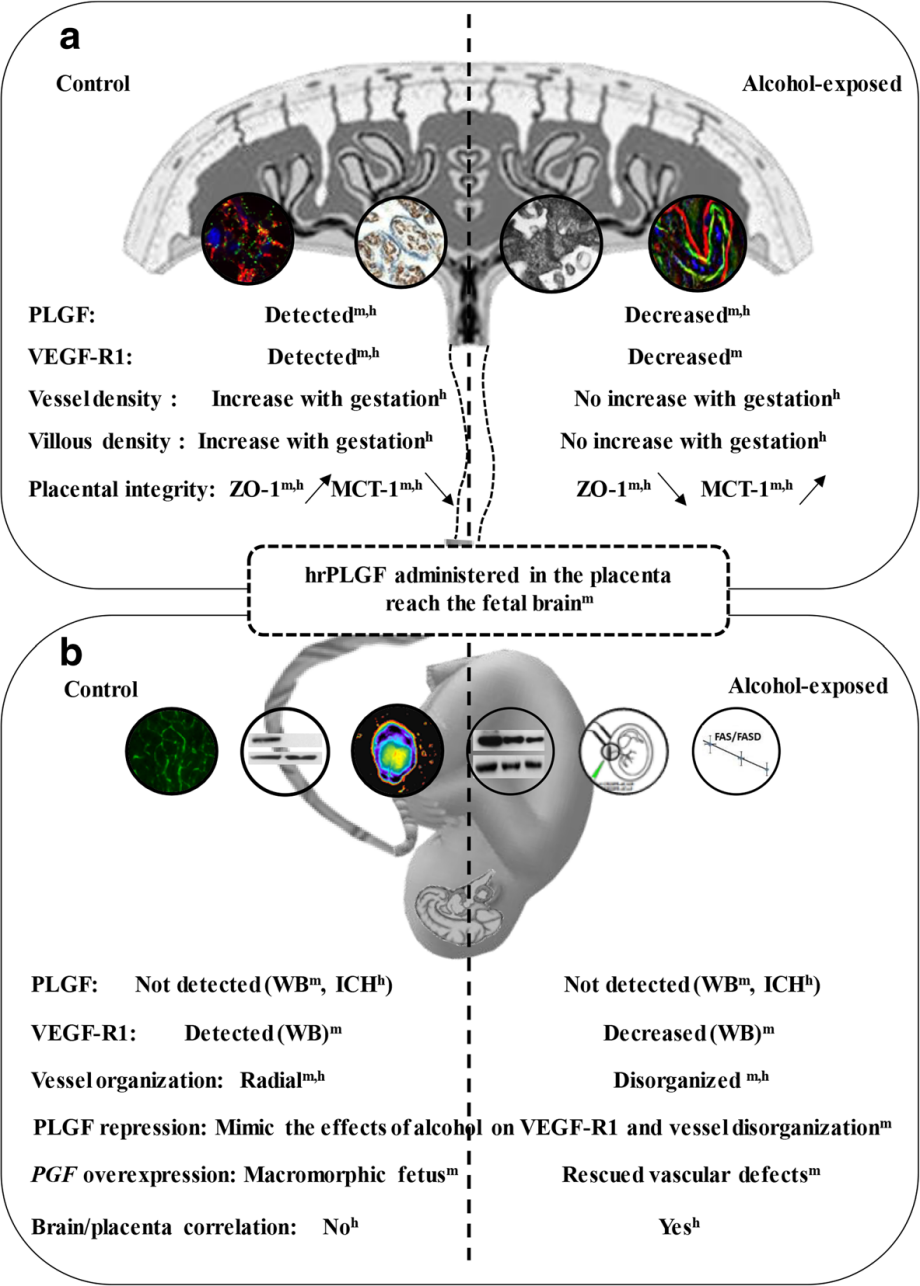
Diagram summarizing the main effects of in utero alcohol exposure on the placenta and the fetal brain in mouse and human. **a** In the placenta, alcohol induced a decrease of PLGF expression in both mouse and human. This effect was associated with a decrease of VEGF-R1 levels in mouse. At a structural level, alcohol consumption altered the density of both villi and vessels in humans. The placental integrity was impacted by a decrease of the placental barrier marker ZO-1 and an increase of the energy metabolism marker MCT-1. **b** In the fetal brain, in utero alcohol exposure induced a disorganization of the cortical vasculature. Cortical VEGF-R1 levels were decreased, whereas PLGF was not detected. Recombinant human PLGF administered in the placenta reached the fetal brain. In utero repression of *PGF* transcription by shRNA mimicked the effects of alcohol on VEGF-R1 in the fetal brain while placental over-expression of the *PGF* gene induced macromorphic fetuses in the control group and rescued the effects of in utero alcohol exposure on vascular defects in the fetal brain. In human, vascular brain defects correlated with vascular placental defects.^m,h^ indicate the experiments performed in mouse and/or human; WB, Western blot approach; IHC, immunohistochemistry

Data on the effect of alcohol on the fetal brain vasculature during pregnancy are scarce [22], but several adult studies indicate that alcohol interacts with angiogenesis [39, 50]. We showed that a transient exposure of the fetus to alcohol during a developmental window in which cranio-facial dysmorphism is not induced [29] can interfere with brain angiogenesis. The critical role of angiogenesis in neurodevelopment is evident [5, 51]. Not only are the guidance molecules used by neurons and endothelial cells to migrate to their final destination similar [5], but the migrating cells closely interact [28, 48]. Regarding in utero alcohol exposure, our data revealed a marked decrease in VEGF-R1 levels in cortical extracts from E20 fetuses (Fig. 7). In addition, PLGF, which binds exclusively to VEGF-R1, is poorly expressed in the fetal brain but is massively synthesized by the placenta [3].

At a mechanistic level, the way in which placental PLGF could act to regulate fetal brain angiogenesis have to be fully investigated. However, several data from the literature would plaid in favor of a direct effect. Indeed, consistent with the present data, it has been recently showed that PLGF is detected in the fetal blood of human neonates [35]. In addition, VEGF-R1 is expressed by tip cells and relative levels of VEGF-R1 and VEGF-R2 contribute in tip cell position [21]. In particular, it has been shown that PLGF may regulate angiogenesis by competing with VEGFA for VEGF-R1 [4]. Alternatively, PLGF might also contribute to the control of angiogenesis by modulating intracellular signals through VEGF-R1 [1].

At a neurodevelopmental level, multiple aspects of central nervous system development can be affected by alcohol exposure, but it has been clearly described abnormalities of neuronal migration as well as corpus callosum defects [41]. Recently, it has been demonstrated that some nervous cell types such as oligodendrocytes [48] and GABA interneurons [51] require a vascular-dependent interaction to migrate. Taken together, these data suggest that abnormal brain vascular development resulting from *PGF* dysfunction may contribute in some alterations described in FASD children such as impaired cell migration.

Maternal ethanol consumption from the beginning of pregnancy is well known to impair placental development. However, while previous transcriptomic studies showed that alcohol altered the expression of angiogenic factors in the placenta of pregnant rats, [43] its impact on the placental vasculature and the VEGF/PLGF system was little investigated in animal models [19] and not described in humans. The placenta appears to be a forgotten organ, although it is surely a promising source of biomarkers [34]. In our series, the analysis of at least 40 placentae per group indicated that, in the control group, a strong increase of vessel density occurred during the last trimester of gestation, consistent with the massive increase of energetic needs of the developing fetal brain (Fig. 7) [9]. In contrast, in the alcohol-exposed group, this increase did not occur and the total lumen area of vessels was markedly reduced. Western blot experiments showed that alcohol induced a marked decrease of PLGF levels in mouse and human placentae. In human, PLGF immunolabeling and protein levels were reduced from midgestation compared with control placentae in which PLGF was present at term, suggesting a role of PLGF in placenta development and maintenance until term. Consistent with this hypothesis, adult knock-out mice invalidated for PLGF have been shown to have a marked reduction of the vessel lumen diameter [4]. Altogether, these data demonstrate for the first time that during human pregnancy alcohol impairs the protein expression of angiogenic markers in the placenta and these molecular defects are associated with marked histomorphometric abnormalities affecting the placental vasculature.

During pregnancy the umbilical cord blood contains PLGF [6, 35]. Our data show that in utero alcohol exposure impairs both the expression of brain VEGF-R1 and the radial organization of cortical microvessels, leading to speculation that PLGF can reach the fetal brain and contributes to alcohol-induced angiogenic impairments. Both trans-UV illumination and ELISA approaches revealed that a fluorescent probe and human PLGF were detectable in the mouse fetal brain 20–30 min after placental microinjection (Fig. 7). Moreover, PLGF was detected by Western blot in the cephalic blood of E20 fetuses while in utero repression of PLGF expression in the placenta significantly reduced VEGF-R1 protein levels and impaired vessel organization in the fetal brain (Fig. 7). Altogether, these data indicate that PLGF expressed in the placenta can reach the fetal brain and mimics the effects of in utero alcohol exposure on VEGF-R1 expression and brain vascular defects.

Alcohol consumption frequently co-occurs with the use of other substances including tobacco or illicit drugs [40]. This point represents a limitation in the interpretation of alcohol-induced effects in human. On another hand, among the numerous animal studies, 80% fail to predict drug effects in human [36]. Dealing with this issue, the strategy which is more and more adopted by research groups is translational medicine [13]. In the present study, most data found in human have been confirmed in our animal model of mono-intoxication with alcohol (Fig. 7) supporting a robust link between alcohol, placental PLGF and brain vascular defects. In addition, overexpression PLGF experiments revealed effects on somatic growth of the fetus opening new research avenues regarding PLGF and in utero growth retardation (IURG) [24].

Most infants with FASD are not diagnosed at birth. A recent cohort study revealed that 86.5% of children or adolescents (4 to 18 years old) with FASD had never been previously diagnosed or had been misdiagnosed [7]. This high rate of missed diagnosis significantly affects therapeutic care, the social integration of the infants and economic costs [38]. In addition, as demonstrated for other pathologies such as autism spectrum disorders, the earlier medical care is started, the better the outcomes probably because of the high plasticity of the nervous system in the first years following birth [27]. The major limitation of the existing biomarkers of alcohol consumption during pregnancy can be summed up in one question: “Was the fetus exposed to alcohol?” [20]. Determining prenatal alcohol exposure is crucial to identify the children/population at risk, but it is not realistic to assess all infants with prenatal alcohol exposure. First, a “safe” dose of alcohol is controversial and highly debated [16, 33]; second, patterns of alcohol consumption differ (chronic/acute) and their effect on the fetus is not the same [10]; and third, the developing brain has windows of vulnerability during which potential harm is greater [25, 49]. These limits also contribute to the discrepancies between different cohort studies on the impact of alcohol consumption on the infant [26, 31, 45]. Thus, the identification of biomarkers of alcohol-induced brain effects after fetal exposure is required. The present study revealed a strong correlation between placental and brain vascular defects in the context of prenatal alcohol exposure. The PLGF levels (<40%) in placentae from women who consumed alcohol during pregnancy appeared to have a predictive value for vascular brain defects. In addition, the demonstration that *PGF* CRISPR-dCas9 activation is able to restore a correct cortical angiogenesis opens new avenues of research regarding a possible prevention of alcohol-induced behavioral troubles. Indeed, as observed in human, several preclinical studies reported neonatal behavioral troubles and long-term deficits in animals exposed in utero to alcohol such as increased motor activity [22, 42]. PLGF assay could help identify infants with brain damage associated with in utero alcohol exposure, thus contributing to an early diagnosis of FASD and prompt intervention. In addition, the present study highlights the necessity to plan a clinical protocol consisting in following both placental PLGF levels at birth and long term behavioral troubles in infants exposed in utero to alcohol. This work was patented (FR1555727 / PCT/EP2016/064480) and (FR1661813).

## Conclusion

The present study provides the first mechanistic and clinical evidence that decreased PLGF levels in the placenta after in utero alcohol exposure are associated to brain angiogenesis defects. Measurement of PLGF levels at birth in the placenta or the fetal blood may serve as a predictive marker for subsequent neurodevelopmental outcomes of exposed fetuses. Compared with the known exposition markers of maternal alcohol intake, this new generation of “effect” biomarkers could facilitate early diagnosis of FASD.

## Additional files

Additional file 1: Table S1. Origin and characteristics of the primary antibodies used for the immunohistochemical and Western blot studies performed in mouse and human tissues. (DOCX 26 kb)

Additional file 2: Table S2. Main clinical and morphological characteristics of human control group for brain studies. (DOCX 17 kb)

Additional file 3: Table S3. Main clinical and morphological characteristics of the alcohol-exposed group of patients for brain studies. (DOCX 21 kb)

Additional file 4: Table S4. Main clinical and morphological characteristics of human placentae from the control group. (DOC 89 kb)

Additional file 5: Table S5. Main clinical and morphological characteristics of human placentae from the alcohol-exposed group. (DOC 131 kb)

Additional file 6: Table S6. Immunohistochemical characteristics of members of the VEGF-PLGF family in human placentae from the “Control” and “Alcohol” groups. (DOCX 25 kb)

Additional file 7: Table S7. Statistical analysis. (DOCX 23 kb)

Additional file 8: Figure S1. Effects of in utero alcohol exposure on morphometric and ultrastructural characteristics of the placenta from GD20 mice. (a) Visualization by Cresyl violet staining of the effect of alcohol exposure on the laminar structuration of the placenta. The placenta is oriented with its maternal side at the top. Note that alcohol induced protrusions of the junctional zone within the labyrinth zone (dotted lines). (b) Visualization of a typical 3D reconstruction of placental protrusions used for morphometric analysis. (c-d) Visualization at low magnification of the giant trophoblast layer from control (c) and alcohol-exposed (d) groups. Arrows indicate giant trophoblasts. Note the typical rectangular shape of this cell type in placentae from the control group whereas in the alcohol-exposed group the trophoblasts present a round shape. (e-h) Images acquired by electron microscopy at moderate (e and f) and high (g and h) magnifications visualizing the morphology of giant trophoblasts and the presence of zonula occludens (arrows) from control (e and g) and alcohol-exposed (f and h) groups. Note a loss of zonula occludens (stars) in alcohol-treated animals. Inserts present in e and f indicate the area visualized at high magnification in g and h, respectively. *d*: maternal decidua; *j*: junctional zone; *l*: labyrinth zone; *tg*: trophoblast giant layer. (i-l) Quantification by morphometric analysis of the effect of alcohol on the number of placental protrusions (i), the length of protrusions (j), the thickness of the Reichert’s membrane (k) and the proportion of round-shape giant trophoblasts in control and alcohol-exposed placentae (l). *p < 0.05; **p < 0.01; ****p < 0.0001 vs the control group using the unpaired t test. (TIFF 15056 kb)

Additional file 9: Figure S2. Effects of in utero alcohol exposure on ZO-1 and MCT-1 expression and visualization of VEGF-R1 in the mouse placenta. (**a, b**) Visualization by immunohistochemistry of the ZO-1 protein in the labyrinth zone of the mouse placenta from the control (**a**) and the alcohol-exposed (**b**) groups. Note that ZO-1 immunolabeling is dotted and clustered (arrows) in the control group whereas it is diffuse in the alcohol-exposed group. Immunoreactivity against the glucose transporter Glut-1 was done to visualize the trophoblast layers. Hoechst was used to label nuclei. (**c**) Double immunolabeling experiment performed with the monocarboxylate and the glucose transporters MCT-1 and Glut-1, respectively in the labyrinth zone of a control placenta. Note that, contrasting with Glut-1, MCT-1 expression is associated with one syncytiotrophoblast layer (maternal side). Hoechst was used to label nuclei. (**d**) Quantification by Western blot of the expression levels of the proteins ZO-1 and MCT-1 in the placentae from control and alcohol-exposed groups. Western blot experiments showed that placentae from alcohol-exposed animals had significantly decreased ZO-1 levels while MCT-1 protein levels were significantly increased. *p < 0.05, **p < 0.01 vs the control group using the unpaired t test. (**e**, **f**) Immunohistochemistry experiments illustrating the distribution of VEGF-R1 (**e**) and Glut-1 (**f**) in the syncytiotrophoblast layers of the mouse placenta. Hoechst was used to visualize nuclei. **(g)** Immunohistochemistry experiments visualizing Glut-1 and PLGF immunoreactivity in the syncytiotrophoblast layers of the mouse placenta. Hoechst was used to visualize nuclei. (h) Visualization by Western blot of PLGF in 100 μg protein extracts from GD20 placenta and E20 brain and in 4 μl plasma from E20 cephalic blood. (TIFF 23009 kb)

Additional file 10: Figure S3. Effects of placental in utero *PGF* overexpression on head and body sizes of E20 fetuses in control and alcohol groups. (**a, b**) Quantification of head (**a**) and body (**b**) sizes in control (NaCl) et alcohol groups. In a same uterine horn some placentae were not electroporated (black bars), electroporated with control CRISPR-Cas9 plasmids (grey bars) or electroporated with *PGF* CRISPR-dCas9 plasmids (white bars). ##*p* < 0.01; ###*p* < 0.001; ####*p* < 0.0001 vs the control group and *p < 0.05; **p < 0.01 as indicated using the two way ANOVA test followed by Tukey’s post hoc test. (TIFF 7601 kb)

Additional file 11: Figure S4. Histomorphometric characterization of the effects of in utero alcohol exposure on human placentae from WG20 to WG25. (**a, b)** Immunohistochemistry performed against CD31 and toluidine blue counterstaining visualizing microvessels (brown) present in placental villi (blue) from control and alcohol-exposed groups collected at gestational ages ranging from [20–25 WG]. (**c**) Percentage of villi classified by sizes in placentae from control and alcohol-exposed groups collected at gestational ages ranging from [20–25 WG].*p < 0.05 vs the control group using the unpaired t test (**d**) Repartition of vessels per size of villi in placentae from control and alcohol-exposed groups collected at gestational ages ranging from [20–25 WG]. (**e**) Luminal vascular area per size of villi in placentae from control and alcohol-exposed groups collected at gestational ages ranging from [20–25 WG]. (TIFF 19794 kb)

Additional file 12: Figure S5. Histomorphometric characterization of the effects of in utero alcohol exposure on human placentae from WG25 to WG35. (**a**, **b**) Immunohistochemistry performed against CD31 and toluidine blue counterstaining visualizing microvessels (brown) present in placental villi (blue) from control and alcohol-exposed groups collected at gestational ages ranging from [25–35 WG]. (**c**) Percentage of villi classified by sizes in placentae from control and alcohol-exposed groups collected at gestational ages ranging from [25–35 WG]. *p < 0.05 vs the control group using the unpaired t test. (**d**) Repartition of vessels per size of villi in placentae from control and alcohol-exposed groups collected at gestational ages ranging from [25–35 WG]. *p < 0.05 vs the control group using the unpaired t test. (**e**) Luminal vascular area per size of villi in placentae from control and alcohol-exposed groups collected at gestational ages ranging from [25–35 WG]. *p < 0.05 vs the control group using the unpaired t test. (TIFF 19271 kb)

Additional file 13: Figure S6. PlGF immunoreactivity of the main placental compartments in control and prenatally alcohol-exposed neonates for gestational ages [35–42 WG]. (a, b) Strongly immunoreactive decidual cells in a control placental maternal floor (arrow), conversely to those of a prenatally alcohol-exposed neonate, where decidual cells exhibit a weak immunoreactivity (arrow). (c, d) Circulating PlGF in the villous capillaries (arrow) in a normal placenta at term, contrasting with absent intra-luminal PlGF immunoreactivity in the villous vessels of a prenatally ethanol-exposed neonate (arrow). (e, f) Strong PlGF immunoreactivity of the villous syncytiotrophoblasts in a control placenta at term (arrow), contrasting with weak and irregular PlGF immunoreactivity in the villous trophoblasts in a prenatally ethanol-exposed neonate (arrow). (TIFF 25948 kb)

## Abbreviations

PLGF: placental growth factor
VEGF-R1: vascular endothelial growth factor receptor 1
PGF: placental growth factor gene
