# Supplementary material for: PLGF, a placental marker of fetal brain defects after in utero alcohol exposure
Source: Acta Neuropathol Commun. 2017 Jun 6;5:44. doi: 10.1186/s40478-017-0444-6 (PMC5461764; doi:10.1186/s40478-017-0444-6)
Supplement: Supplementary file 1 — Origin and characteristics of the primary antibodies used for the immunohistochemical and Western blot studies performed in mouse and human tissues. (DOCX 26 kb) [file 40478_2017_444_MOESM1_ESM.docx]

**Table S1** Origin and characteristics of the primary antibodies used for the immunohistochemical and Western blot studies performed in mouse and human tissues

| **Antibodies** | **Trade reference** | **Purified species** | **Species detection origin** | **Supplier** | **Dilution** | **Solution of incubation** |
| --- | --- | --- | --- | --- | --- | --- |
| PECAM /CD31 | WB: (M-20) sc-1506 | Goat | Mouse, rat, human | Santa Cruz | 1/500 | BSA (5% in TBST) |
|  | Mouse IHC: cat 550274 | Rat | Mouse | BD-Pharmigen | 1/400 | BSA (1% in PBS) |
|  | Human IHC  CD31-1A10-CE | Mouse | Human | LEICA | 1/50 | 0032-18 antibody diluant reagent solution  Invitrogen |
| PLGF | WB (mouse):(M-18) sc-1882  ab 9542 | Goat  Rabbit | Mouse, rat  Mouse/human | Santa Cruz  abcam | 1/1000  1/1000 | Milk (5% in TBST) |
|  | WB (human):  ab 9542 | Rabbit | Mouse/human | abcam | 1/400 | 0032-18 antibody diluant reagent solution  Invitrogen |
|  | IHC (human): (C-20) sc-1880 | Rabbit | Mouse, human | abcam | 1/1000 | Milk (5% in TBST) |
| VEGFA | WB: (A-20) sc-152 | Rabbit | Human, mouse, rat | Santa Cruz | 1/500 | BSA (5% in TBST) |
|  | Human IHC: (A-20) sc-152 | Rabbit | Human, mouse, rat | Santa Cruz | 1/50 | 0032-18 antibody diluant reagent solution  Invitrogen |
| VEGF-R1/Flt-1 | WB: (C-17) sc-316 | Rabbit | Mouse, rat, human | Santa Cruz | 1/1000 | Milk (5% in TBST) |
|  | Mouse IHC: ab32152 | Rabbit | Mouse, rat, human | abcam | 1/400 | BSA (1% in PBS) |
|  | Human IHC: (A-20) sc-152 | Rabbit | Mouse, rat, human | abcam | 1/100 | 0032-18 antibody diluant reagent solution  Invitrogen |
| VEGF-R2/Flk-1 | WB: (N-931) sc-505 | Rabbit | Mouse, rat, human | Santa Cruz | 1/1000 | Milk (5% in TBST) |
|  | Mouse IHC: ab9530 | Mouse | Rat, sheep, human | abcam | 1/200 | BSA (1% in PBS) |
|  | Human IHC: sc-6251 | Mouse | Mouse, rat, human | Santa Cruz | 1/200 | 0032-18 antibody diluant reagent solution  Invitrogen |

| **Antibodies** | **Trade reference** | **Purified Species** | **Species detection origin** | **Supplier** | **Dilution** | **Solution of incubation** |
| --- | --- | --- | --- | --- | --- | --- |
| ZO-1 | WB and IHC: ab59720 | Rabbit | Mouse, chicken, human | abcam | 1/1000 | BSA (1% in PBS) |
| Glut-1 | Mouse IHC: 07-1401 | Rabbit | Human, mouse, rat | Millipore Life science | 1/600 | BSA (1% in PBS) |
|  | IHC sc-1605  Human IHC: A3536 | Goat  Rabbit | Human, mouse, rat  Human | Santa Cruz  Dakopatts | 1/400  1/100 | BSA (1% in PBS)  0032-18 antibody diluant reagent solution  Invitrogen |
| MCT-1 | WB: (T-19) sc-14917 | Goat | Mouse, rat, human | Santa Cruz | 1/1000 | Milk (5% in TBST) |
|  | IHC: AB1286-I | Chicken | Rat, mouse | Millipore Life science | 1/600 | BSA (1% in PBS) |
| β-Actin | WB: A5441 | Mouse | Mouse, chicken, Rabbit, rat, human | Sigma- Aldrich | 1/5000 | Milk (5% in TBST) |
