# Supplementary material for: PLGF, a placental marker of fetal brain defects after in utero alcohol exposure
Source: Acta Neuropathol Commun. 2017 Jun 6;5:44. doi: 10.1186/s40478-017-0444-6 (PMC5461764; doi:10.1186/s40478-017-0444-6)
Supplement: Supplementary file 2 — Main clinical and morphological characteristics of human control group for brain studies. (DOCX 17 kb) [file 40478_2017_444_MOESM2_ESM.docx]

**Table S2** Main clinical and morphological characteristics of human control group for brain studies

| **Case** | **WG** | **Medical termination** | **Cerebral**  **maturation**  **(WG)** | **Cause of death** |
| --- | --- | --- | --- | --- |
| **number** |  | **of pregnancy** |  |  |
| **1** | **19** | **Yes** | **19** | **Trisomy 21 No malformations** |
| **2** | **20** | **No** | **20** | **Diamnionic-dichorionic twin pregnancy**  **Premature rupture of the membranes** |
| **3** | **22** | **No** | **22** | **Diamnionic-dichorionic twin pregnancy**  **Acute chorioamnionitis** |
| **4** | **22** | **No** | **22** | **Abruptio placentae**  **Premature rupture of the membranes** |
| **5** | **30** | **No** | **30** | **Neonatal septicemia (*Escherichia coli*)** |
| **6** | **31** | **No** | **31** | **Hypovolemic collapse** |
| **7** | **38** | **No** | **41** | **Autosomal recessive polycystic kidney disease** |

Fetal biometry according to Guihard-Costa & Larroche (1990) [16] and Feess-Higgins & Larroche (1987) [10]. WG, weeks of gestation.
