# Supplementary material for: PLGF, a placental marker of fetal brain defects after in utero alcohol exposure
Source: Acta Neuropathol Commun. 2017 Jun 6;5:44. doi: 10.1186/s40478-017-0444-6 (PMC5461764; doi:10.1186/s40478-017-0444-6)
Supplement: Supplementary file 3 — Main clinical and morphological characteristics of the alcohol-exposed group of patients for brain studies. (DOCX 21 kb) [file 40478_2017_444_MOESM3_ESM.docx]

**Table S3** Main clinical and morphological characteristics of the alcohol-exposed group of patients for brain studies

| **Case number** | **WG** | **Cause of death** | **Maternal alcohol intake** | **Maternal**  **Comorbidity** | **IUGR (<3rd percentile)** | **Brain**  **(%ile)** |
| --- | --- | --- | --- | --- | --- | --- |
| **1** | **20** | **TOP for**  **Maternal distress** | **Chronic alcohol intake and binge drinking*** | **HIV, Hepatitis C**  **Multi-drug addiction** | **10th** | **39.30 g (25th)**  **No brain malformation** |
| **2** | **21** | **IUFD**  **Chorioamnionitis** | **Chronic alcohol intake**** | **No** | **No** | **51.75 g (50th)**  **No brain malformation** |
| **3** | **21** | **IUFD**  **Premature rupture of the membranes, twin-twin transfusion syndrome** | **Chronic alcohol intake**** | **Increased MGV** | **3rd** | **61.8 g (50th)**  **No brain malformation** |
| **4** | **21** | **IUFD Premature rupture of the membranes, twin-twin transfusion syndrome** | **Chronic alcohol intake**** | **Increased MGV** | **3rd** | **56.1g (50th)**  **No brain malformation** |
| **5** | **22** | **TOP for Maternal distress** | **Chronic alcohol intake (262 g/day) + Psychotrops*** | **Psychotic Disorder**  **Increased MGV** | **3rd** | **63.5 g (50th)**  **No brain malformation** |
| **6** | **22** | **TOP for Maternal distress** | **Daily chronic alcohol intake*** | **Multi-drug addiction**  **Sodium Valproate (epilepsy)** | **50th** | **45.65 g (3rd)**  **Microcephaly**  **Arnold Chiari malformation type 2**  **Sacral agenesis spina bifida** |
| **7** | **29** | **IUFD**  **Polymalformative syndrome** | **Daily chronic alcohol intake*** | **Increased MGV and γGT** | **5th** | **178 g (5th)**  **No brain malformation** |
| **8** | **30** | **IUFD**  ***Abruptio placentae*** | **Daily chronic alcohol intake**** | **Cannabis addiction**  **Treated hypothyroidism** | **10th** | **211 g (50th)**  **Bilateral intraventricular cerebral haemorrhage** |
| **9** | **31** | **IUFD**  **Preeclampsia** | **Daily chronic alcohol intake*** | **Increased MGV and γGT** | **3rd** | **197.05 g (<3rd)**  **Microcephaly**  **No brain malformation** |
| **10** | **33** | **IUFD**  **Acute alcohol intoxication** | **Chronic and binge drinking***  **(4.98 g/L)** | **Multi-drug addiction**  **Increased MGV and γGT**  **First pregnancy: IUFD at 33 WG)**  **One child alive with FAS.** | **50th** | **348.15 g (50th)**  **No brain malformation** |
| **11** | **38** | **IUFD** | **Chronic and binge drinking**** | **Heroin addiction** | **25th** | **285 g (<3rd)**  **No brain malformation** |

Fetal biometry according to Guihard-Costa & Larroche (1990) [16] and Feess-Higgins & Larroche (1987) [10]. WG, weeks of gestation; TOP, medical termination of the pregnancy; IUFD, in utero fetal death; IUGR, intrauterine growth retardation; CNS, central nervous system; MGV, mean globular volume; γGT, gamma-glutamyl transferase; * maternal self report; ** suspected.
