# Supplementary material for: PLGF, a placental marker of fetal brain defects after in utero alcohol exposure
Source: Acta Neuropathol Commun. 2017 Jun 6;5:44. doi: 10.1186/s40478-017-0444-6 (PMC5461764; doi:10.1186/s40478-017-0444-6)
Supplement: Supplementary file 4 — Main clinical and morphological characteristics of human placentae from the control group. (DOC 89 kb) [file 40478_2017_444_MOESM4_ESM.doc]

**Table S4** Main clinical and morphological characteristics of human placentae from the control group

| **Case**  **number** | **Maternal age** | **Term, WG** | | **IUFD, TOP, alive** | | **Fetal or neonatal weight** | | | **Postmortem lesions** | | | | **Placental weight (%ile)** | | | **Placental lesions** | | | **Technical investigations** | | |  | |
| --- | --- | --- | --- | --- | --- | --- | --- | --- | --- | --- | --- | --- | --- | --- | --- | --- | --- | --- | --- | --- | --- | --- | --- |
| **1** | **35** | **21** | | **TOP** | | **243 g**  **(5th)** | | | **Trisomy 18**  **omphalocele** | | | | **47 g**  **(< 3th)** | | | **No** | | | **Morphometry**  **IHC** | | |  | |
| **2** | **24** | **21** | | **Dead at birth** | | **372 g**  **(50th)** | | | **No autopsy** | | | | **138 g (50th)** | | | **Abruptio placentae** | | | **Morphometry**  **IHC** | | |  | |
| **3** | **30** | **22** | | **Dead at birth** | | **480 g**  **(75th)** | | | **No autopsy** | | | | **130 g (25th)** | | | **Premature rupture of the membranes, acute chorioamniotitis** | | | **Morphometry**  **IHC** | | |  | |
| **4** | **27** | **23** | | **TOP** | | **NA** | | | **Myelomeningocele**  **Arnold-Chiari malformation** | | | | **152 g (25th)** | | | **No** | | | **Morphometry**  **IHC** | | |  | |
| **5** | **41** | **23** | | **TOP** | | **640 g**  **(75th)** | | | **Isolated occipital encephalocele** | | | | **215 g**  **(75th)** | | | **No** | | | **Morphometry**  **IHC** | | |  | |
| **6** | **38** | **24** | | **TOP** | | **220 g**  **(<5th)** | | | **Right ventricular heart hypoplasia** | | | | **165 g (25th)** | | | **No** | | | **Morphometry**  **IHC** | | |  | |
| **7** | **30** | **24** | | **TOP** | | **NA** | | | **No autopsy** | | | | **260 g**  **(>90th)** | | | **Abruptio placentae acute chorioamniotitis** | | | **Morphometry**  **IHC** | | |  | |
| **8** | **21** | **25** | | **Diamnionic dichorial pregnancy Dead at birth** | | **NA** | | | **No autopsy** | | | | **307 g**  **(>90th)** | | | **Premature rupture of the membranes** | | | **Morphometry**  **IHC** | | |  | |
| **9** | **31** | **27** | | **Dead at birth** | | **1060 g**  **(5th)** | | **No autopsy** | | | | **330 g (90th)** | | | **Acute chorioamniotitis** | | | **Morphometry**  **IHC** | | |  | | |
| **10** | **22** | **27** | | **TOP** | | **NA** | | **Tanatophoric dysplasia**  **No autopsy** | | | | **227 g (50th)** | | | **No** | | | **Morphometry**  **IHC** | | |  | | |
| **11** | **32** | **28** | | **Alive** | | **1260 g**  **(50th)** | |  | | | | **250 g**  **(25th)** | | | **Premature rupture of the membranes** | | | **Morphometry**  **IHC** | | |  | | |
| **12** | **31** | **29** | | **TOP** | | **1390 g**  **(50th)** | | **Corpus callosum agenesis and ventricular dilatation** | | | | **238 g (50th)** | | | **No** | | | **Morphometry**  **IHC** | | |  | | |
| **13** | **31** | **29** | | **Alive** | | **NA** | |  | | | | **280 g (75th)** | | | **Premature rupture of the membranes** | | | **Morphometry**  **IHC** | | |  | | |
| **14** | **30** | **29** | | **Alive** | | **1300 g**  **(50th)** | |  | | | | **270 g (5th)** | | | **Abruptio placentae** | | | **Morphometry**  **IHC** | | |  | | |
| **15** | **26** | **29** | | **Alive** | | **NA** | |  | | | | **325 g (75th)** | | | **Acute chorioamniotitis** | | | **Morphometry**  **IHC** | | |  | | |
| **16** | **31** | **30** | | **Diamnionic dichorial pregnancy** | | **1260 g**  **(5th)**  **1100 g**  **(5th)** | |  | | | | **204 g (25th)** | | | **Marginal hematoma** | | | **Morphometry**  **IHC** | | |  | | |
| **17** | **23** | **31** | | **Alive** | | **1450 g**  **(25th )** | |  | | | | **220 g (25th)** | | | **Abruptio placentae** | | | **Morphometry**  **IHC** | | |  | | |
| **18** | **30** | **31** | | **Alive** | | **1730 g**  **(50th)** | |  | | | | **320 g**  **(50th)** | | | **No** | | | **Morphometry**  **IHC** | | |  | | |
| **Table S4 (continued)** | | | | | | | | | |  | | | | | | | | | | | | | |
| **Case**  **number** | **Maternal age** | **Term** | | **IUFD, TOP, alive** | | **Fetal or neonatal weight** | | | **Postmortem lesions** | | | | **Placental weight (%ile)** | | | **Placental lesions** | | | **Technical investigations** | | |  | |
|  |  |  |  | |  | |  | | | |  | | |  | | |  | | |  | | |  |
| **19** | **30** | **32** | | **TOP** | | **1383 g**  **(25th)** | | **Pontocerebellar hypoplasia type V** | | | | **350 g**  **(50th)** | | | **No** | | | **Morphometry**  **IHC** | | |  | | |
| **20** | **19** | **32** | | **TOP pulmonary artery hypoplasia** | | **1280 g**  **(5th)** | | **Heart malformation Obstrucutive uropathy** | | | | **205 g (5th)** | | | **No** | | | **Morphometry**  **IHC** | | |  | | |
| **21** | **30** | **33** | | **Alive** | | **1960 g**  **(50th)** | |  | | | | **425 g**  **(25th)** | | | **No** | | | **Morphometry**  **IHC** | | |  | | |
| **22** | **25** | **33** | | **Alive** | | **NA** | |  | | | | **331 g**  **(25th)** | | | **Premature rupture of the membranes** | | | **Morphometry**  **IHC** | | |  | | |
| **23** | **35** | **33** | | **Alive** | | **NA** | |  | | | | **320 g (50th)** | | | **Acute chorioamniotitis** | | | **Morphometry**  **IHC** | | |  | | |
| **24** | **29** | **34** | | **Alive** | | **2760 g**  **(50th)** | |  | | | | **427 g**  **(50th–75th)** | | | **No** | | | **Morphometry**  **IHC** | | |  | | |
| **25** | **26** | **36** | | **TOP** | | **2837 g**  **(50th)** | | **Tuberous sclerosis** | | | | **405 g**  **(25 th)** | | | **No** | | | **Morphometry**  **IHC** | | |  | | |
| **26** | **37** | **37** | | **Alive** | | **2780 g**  **(50th)** | |  | | | | **543 g**  **(75 th)** | | | **No** | | | **Morphometry**  **IHC** | | |  | | |
| **27** | **27** | **37** | | **Alive** | | **2680 g**  **(50th)** | |  | | | | **469 g**  **(50 th)** | | | **No** | | | **Morphometry**  **IHC** | | |  | | |
| **28** | **25** | **37** | | **Alive** | | **3630 g**  **(95th)** | |  | | | | **432 g**  **(25 th)** | | | **Abruptio placentae** | | | **Morphometry**  **IHC WB** | | |  | | |
| **29** | **26** | **38** | | **Alive** | | **2280 g**  **(25th)** | |  | | | | **375 g**  **(<10th)** | | | **No** | | | **Morphometry**  **IHC** | | |  | | |
| **30** | **26** | **38** | | **Alive** | | **3490 g**  **(95th)** | |  | | | | **605 g (90th)** | | | **No** | | | **Morphometry**  **IHC** | | |  | | |
| **31** | **38** | **38** | | **Alive** | | **2530 g**  **(25th)** | |  | | | | **385 g (10th)** | | | **Placenta prævia** | | | **Morphometry**  **IHC** | | |  | | |
| **32** | **28** | **39** | | **Alive** | | **3700 g**  **(95th)** | |  | | | | **510 g**  **(50th)** | | | **No** | | | **Morphometry**  **IHC** | | |  | | |
| **33** | **32** | **39** | | **Alive** | | **3230 g**  **(50th)** | |  | | | | **412 g**  **(10th–25th)** | | | **No** | | | **Morphometry**  **IHC WB** | | |  | | |
| **34** | **28** | **40** | | **Alive** | | **2840 g**  **(25th)** | |  | | | | **497 g**  **(25th)** | | | **No** | | | **Morphometry**  **IHC WB** | | |  | | |
| **35** | **20** | **40** | | **Alive** | | **3640 g**  **(50th)** | |  | | | | **505 g**  **(50th)** | | | **No** | | | **Morphometry**  **IHC WB** | | |  | | |
| **36** | **38** | **40** | | **Alive** | | **3280 g**  **(50th)** | |  | | | | **480 g**  **(25th)** | | | **No** | | | **Morphometry**  **IHC WB** | | |  | | |
| **37** | **31** | **40** | | **Alive** | | **3220 g**  **(50th)** | |  | | | | **522 g**  **(25th–50th)** | | | **No** | | | **Morphometry**  **IHC** | | |  | | |
| **38** | **19** | **41** | | **Alive** | | **3500 g**  **(50th)** | |  | | | | **425 g**  **(<10th)** | | | **No** | | | **Morphometry**  **IHC WB** | | |  | | |

**Table S4 (continued)**

| **Case**  **number** | **Maternal age** | **Term, WG** | **IUFD, TOP, alive** | **Fetal or neonatal weight** | | **Postmortem lesions** | | **Placental weight** | | **Placental lesions** | | **Technical investigations** | |
| --- | --- | --- | --- | --- | --- | --- | --- | --- | --- | --- | --- | --- | --- |
| **39** | **29** | **41** | **Alive** | **3360 g**  **(50th)** |  | | **450 g (28th)** | | **No** | | **Morphometry**  **IHC** | |  |
| **40** | **39** | **41** | **Alive** | **3810 g**  **(75th)** |  | | **702 g**  **(>90th)** | | **No** | | **Morphometry**  **IHC** | |  |
| **41** | **31** | **42** | **Alive** | **NA** |  | | **540 g**  **(50th)** | | **No** | | **Morphometry**  **IHC WB** | |  |

Fetal biometry according to Guihard-Costa et al (2002) [17] and Pinar et al (1996) [35]. IHC, immunohistochemistry; IUFD, intrauterine fetal death; NA, not available; TOP, medical termination of the pregnancy; WB, Western blot; WG, weeks of gestation.
