# Supplementary material for: PLGF, a placental marker of fetal brain defects after in utero alcohol exposure
Source: Acta Neuropathol Commun. 2017 Jun 6;5:44. doi: 10.1186/s40478-017-0444-6 (PMC5461764; doi:10.1186/s40478-017-0444-6)
Supplement: Supplementary file 5 — Main clinical and morphological characteristics of human placentae from the alcohol-exposed group. (DOC 131 kb) [file 40478_2017_444_MOESM5_ESM.doc]

**Table S5** Main clinical and morphological characteristics of human placentae from the alcohol-exposed group

| **Case**  **number** | **Maternal age** | **Gravidity/parity** | **Medical history** | **Alcohol consumption and others** | **Term, WG** | **IUFD, TOP, alive** | | | **Fetal or neonatal weight (%ile)** | **General autopsy findings** | **Placental weight**  **(%ile)** | **Placental lesions** | **Brain weight and lesions**  **(%ile)** | **Technical investigations** |
| --- | --- | --- | --- | --- | --- | --- | --- | --- | --- | --- | --- | --- | --- | --- |
| **1** | **32** | **G8P3** | **Hepatitis C** | **Binge and chronic Tobacco Multiple addictions** | **20** | **TOP for maternal distress** | | | **305 g (25th)** | **No visceral malformation** | **85 g (<3rd )** | **No lesion** | **39.30 g (25 th)**  **No brain malformation** | **Morphometry**  **IHC** |
| **2** | **40** | **G3P1** | **Epilepsy**  **Anorexia** | **Chronic Cannabis Tobacco** | **21** | **TOP for maternal distress** | | | **391 g (50th)** | **FAS dysmorphism**  **No visceral malformation** | **120 g (10th)** | **No lesion** | **63.20 g (50 th)**  **No brain malformation** | **Morphometry**  **IHC** |
| **3** | **32** | **G2P0** | **Diamnionic dichorial pregnancy** | **Suspected chronic**  **Increased MGV at 98** | **21** | **Spontaneous abortion**  **Premature rupture of the membranes** | | | **321 g (50th)** | **FAS dysmorphism Cystic adenoid malformation of the lungs** | **250 g (25th)** | **Twin-twin transfusion Chorioamniotitis** | **61.8 g (50th)**  **No brain malformation** | **Morphometry**  **IHC** |
| **4** | **32** | **G2P0** | **Diamnionic dichorial pregnancy** | **Suspected chronic** | **21** | **Spontaneous abortion**  **Premature rupture of the membranes** | | | **283 g (5th)** | **FAS dysmorphism**  **No visceral malformation** | **250 g (25th)** | **Twin-twin transfusion Chorioamniotitis** | **56.1 g (50th)**  **No brain malformation** | **Morphometry**  **IHC** |
| **5** | **30** | **G5P3**  **1 spontaneous**  **abortion** |  | **Suspected Chronic**  **High γGT Tobacco** | **21** | **Suspicion of chorioamniotitis** | | | **288 g (50th)** | **No visceral malformation** | **140 g (50th)** | **Marginal hematoma** | **51.71 g (50th)**  **No brain malformation** | **Morphometry**  **IHC** |
| **6** | **38** | **G1P0**  **1 spontaneous abortion** | **IUGR**  **Epilepsy**  **Valproic acid Psychotic disorder** | **Chronic**  **Not quantified Increased MGV at 104** | **22** | **TOP for myelomeningocele** | | | **377 g (5th)** | **Hydronephrosis** | **138 g (50th)** | **Excess of fibrin deposit** | **45.65 g (3rd)**  **Microcephaly**  **Arnold Chiari malformation type 2**  **Sacral agenesis spina bifida** | **Morphometry**  **IHC** |
| **7** | **38** | **NA** |  | **Chronic not quantified Tobacco cannabis** | **25** | **Spontaneous abortion at home** | | | **680 g (25th)** | **FAS dysmorphism Left hydronephrosis with ureteral stenosis** | **180 g (25th))** | **Chorioamniotitis Funiculitis** | **133.6 g (50th)**  **Bilateral subependymal hemorrhage**  **No brain malformation** | **IHC** |
| **Table S5 (continued)** | | | | | | |  |  | | | | | | |
| **Case**  **number** | **Maternal age** | **Gravidity/parity** | **Medical history** | **Alcohol consumption and others** | **Term, WG** | **IUFD, TOP, alive** | | | **Fetal or neonatal weight (%ile)** | **General autopsy findings** | **Placental weight**  **(%ile)** | **Placental lesions** | **Brain weight and lesions**  **(%ile)** | **Technical investigations** |
| **8** | **36** | **NA** |  |  | **25** | **Alive** | | | **800 g (50th)** |  | **167 g (50th)** | **Chorioamniotitis Funiculitis** |  | **IHC** |
| **9** | **38** | **NA** | **IUGR** | **Chronic 35 g/d** | **29** | **TOP for polymalformative syndrome** | | | **879 g (<3rd)** | **FAS dysmorphism Fallot tetralogy** | **130 g (<3rd)** | **No lesion** | **178 g (5th)**  **No brain malformation** | **Morphometry**  **IHC** |
| **10** | **45** | **NA** | **Caesarian section for preeclampsia** | **30 g/d** | **29** | **Alive** | | | **800 g (<3rd)** |  | **145 g (<3rd)** | **Decidual vasculopathy** |  | **IHC** |
| **11** | **31** | **NA** |  |  | **29** | **TOP for corpus callosum agenesis** | | | **1390 g (50th)** | **No malformation** | **238 g (50th)** | **No lesion** | **205.8 g (50th)**  **Corpus callosum agenesis** | **IHC** |
| **12** | **39** | **NA** | **Hypothyroidism** | **Chronic Cannabis Tobacco** | **30** | **IUFD** | | | **1317 g (50th)** | **No malformation** | **340 g (50th)** | **Multiple infarcts Abruptio placentae** | **211 g (50th)**  **Bilateral intraventricular cerebral haemorrhage** | **Morphometry**  **IHC** |
| **13** | **34** | **G5P0** | **Preeclampsia** | **Chronic**  **Not quantified**  **High γGT concentration MGV at 103** | **31** | **IUFD** | | | **1100 g (<3rd)** | **No lesion** | **150 g (<3rd)** | **Abruptio Placentae** | **197.05 g (<3rd)**  **Microcephaly**  **No brain malformation** | **Morphometry**  **IHC** |
| **14** |  | **NA** |  | **Chronic**  **Not quantified** | **31** | **Alive** | | | **1600 g (50th)** | **No lesion** | **365 g (25th)** | **No lesion** | **Cystic periventricular leukomalacia Microcephaly** | **Morphometry**  **IHC** |
| **15** | **38** | **G3P2** | **One previous child with FAS** | **Severe chronic and binge drinking**  **(4.98 g/L)** | **33** | **IUFD** | | | **2485 g (90th)** | **Aspiration pneumoniae** | **400 g (50th–75th)** | **Infarcts (<20%)** | **348.15 g (50th)**  **No brain malformation** | **Morphometry**  **IHC** |
| **16** | **38** | **NA** | **Emergency caesarian section for severe bleeding** | **Chronic**  **Not quantified** | **33** | **Alive** | | | **2040 g (50th)** |  | **320 g (50th )** | **Premature involution of the placenta** |  | **Morphometry**  **IHC** |
| **Table S5 (continued)** | | | | | | |  |  | | | | | | |

| **Case**  **number** | **Maternal age** | | | | **Gravidity/parity** | | | **Medical history** | | **Alcohol consumption and others** | | **Term, WG** | | | **IUFD, TOP, alive** | | | **Fetal or neonatal weight (%ile)** | | | **General autopsy findings** | | | **Placental weight**  **(%ile)** | | | **Placental lesions** | | **Brain weight and lesions**  **(%ile)** | | | | **Technical investigations** | | |
| --- | --- | --- | --- | --- | --- | --- | --- | --- | --- | --- | --- | --- | --- | --- | --- | --- | --- | --- | --- | --- | --- | --- | --- | --- | --- | --- | --- | --- | --- | --- | --- | --- | --- | --- | --- |
| **17** | **33** | | | **NA** | | | **Cesarian section for severe IUGR** | | | **Chronic**  **Not quantified Tobacco** | | | **34** | | | **Alive** | | **NA** | |  | | | **330 g (10th)** | | | **Premature involution of the placenta** | | | |  | | **IHC** | | |  |
| **18** | **29** | | | **NA** | | | **Premature spontaneous delivery** | | | **Chronic**  **Not quantified** | | | **34** | | | **Alive** | | **NA** | |  | | | **305 g (5th)** | | | **Premature involution of the placenta** | | | |  | | **Morphometry**  **IHC** | | |  |
| **19** | **39** | | | **NA** | | |  | | | **Chronic**  **Not quantified** | | | **35** | | | **Alive** | | **NA** | | **FAS dysmorphism** | | | **530 g (95th)** | | | **No lesion** | | | |  | | **IHC WB** | | |  |
| **20** | **20** | | | **G1P0** | | |  | | | **Regular binge drinking (3 per week)** | | | **35** | | | **Alive** | | **1880 g (5th)** | | **FAS dysmorphism** | | | **375 g (10th–25th)** | | | **No lesion** | | | |  | | **Morphometry**  **IHC** | | |  |
| **21** | **34** | | | **NA** | | | **Diamnionic dichorial pregnancy** | | | **Chronic 40 g/day Binge drinking 50 g/day** | | | **36** | | | **Alive**  **Alive** | | **1700 g (<3rd)**  **1410 g (<3rd)** | |  | | | **265 g (<3rd)** | | | **Chronic hypoxia** | | | |  | | **IHC WB** | | |  |
| **22** | **23** | | | **NA** | | | **Hepatitis C** | | | **Multiple drug addiction**  **Methadone** | | | **37** | | | **Alive** | | **NA** | |  | | | **367 g (5th)** | | | **Chronic hypoxia** | | | |  | | **Morphometry**  **IHC** | | |  |
| **23** | **26** | | | **G2P0** | | |  | | | **Chronic**  **Not quantified Polytoxicomania Tobacco** | | | **37** | | | **IUFD** | | **2450 g (25th)** | |  | | | **390 g (10th)** | | | **Fibrosis of the villi**  **Chronic hypoxia** | | | | **248 g (<3rd)**  **Microcephaly Neuronal ectopias in the white matter** | | **Morphometry**  **IHC** | | |  |
| **24** | **23** | | | **G1P0** | | | **Diamnionic dichorial pregnancy** | | | **Chronic 30 g/day Methadone** | | | **37** | | | **Alive I** | | **NA** | | **FAS dysmorphism** | | | **390 g (10th)** | | | **Marginal hematoma** | | | |  | | **Morphometry**  **IHC** | | |  |
| **25** | **40** | | | **NA** | | | **Not followed pregnancy** | | | **Chronic 100 g/day** | | | **37** | | | **Alive** | | **2055 g (<3rd)** | |  | | | **310 g (<3rd)** | | | **Chronic hypoxia**  **of the villi** | | | |  | | **Morphometry**  **IHC** | | |  |
| **26** | **34** | | | **NA** | | | **IUGR** | | | **Chronic** | | | **37** | | | **Alive** | | **2000 g (<3rd)** | |  | | | **280 g (<3rd)** | | | **Thrombotic vasculopathy** | | | |  | | **Morphometry**  **IHC** | | |  |
| **Case**  **number** | | **Maternal age** | **Gravidity/parity** | | | **Medical history** | | | **Alcohol consumption and others** | | **Term, WG** | | | **IUFD, TOP, alive** | | | **Fetal or neonatal weight (%ile)** | | **General autopsy findings** | | | **Placental weight**  **(%ile)** | | | **Placental lesions** | | | **Brain weight and lesions**  **(%ile)** | | | **Technical investigations** | | |  | |
|  |  | | |  | | |  | | |  | | |  | | |  | |  | |  | | |  | | |  | | | |  | |  | | |  |
| **27** | **25** | | | **NA** | | | **Gestational diabetes** | | | **Chronic**  **Not quantified** | | | **37** | | | **Alive** | | **3570 g (95th)** | |  | | | **650 g (>95th)** | | | **Diabetes placenta Hypertrophy Premature involution** | | | |  | | **Morphometry**  **IHC** | | |  |
| **28** | **43** | | | **G1P1** | | | **Fetal distress Cesarian section** | | | **Chronic**  **Not quantified Increased VGM** | | | **37** | | | **Alive** | | **NA** | |  | | | **345 g (<3rd)** | | | **Premature involution** | | | |  | | **IHC** | | |  |
| **29** | **39** | | | **G3P1** | | | **IUGR** | | | **Chronic**  **Not quantified** | | | **38** | | | **Alive** | | **1940 g (<3rd)** | |  | | | **280 g (<3rd)** | | | **Chronic hypoxia** | | | |  | | **Morphometry**  **IHC** | | |  |
| **30** | **27** | | | **NA** | | |  | | |  | | | **38** | | | **Alive** | | **2680 g (50th)** | |  | | | **254 g (<3rd)** | | | **No lesion** | | | |  | | **IHC WB** | | |  |
| **31** | **22** | | | **G1 P1** | | |  | | | **Multiple addiction 170g/day Tobacco Cannabis Psychotropics** | | | **38** | | | **Alive** | | **1940 g (<3rd)** | |  | | | **415 g (10th)** | | | **Delayed maturation of the placenta** | | | |  | | **Morphometry**  **IHC** | | |  |
| **32** | **40** | | | **G3P2** | | | **IUGR** | | | **Severe chronic and binge drinking 4.8 g/L during labor** | | | **38** | | | **Alive** | | **2180 g (5th)** | |  | | | **285 g (<3rd)** | | | **Villous Hypovascularization** | | | |  | | **Morphometry**  **IHC** | | |  |
| **33** | **39** | | | **NA** | | | **IUGR** | | | **Chronic**  **Not quantified High γGT**  **VGM 103** | | | **38** | | | **Alive** | | **1700 g (<3rd)** | |  | | | **335 g (<3rd)** | | | **Placental hypoxia Premature involution**  **Excess of fibrin deposits** | | | |  | | **Morphometry**  **IHC** | | |  |
| **34** | **42** | | | **NA** | | | **IUGR** | | | **Chronic**  **Not quantified 200 g/day** | | | **38** | | | **Alive** | | **2115 g (<3rd)** | | **FAS dysmorphism** | | | **246 g (<3rd)** | | | **Premature involution** | | | |  | | **Morphometry**  **IHC WB** | | |  |
| **35** | **38** | | | **NA** | | | **UIGR** | | | **Chronic**  **Not quantified** | | | **38** | | | **Alive** | | **2530 g (25th)** | |  | | | **385 g (<3rd)** | | | **Premature involution** | | | |  | | **IHC WB** | | |  |
| **36** | **33** | | | **G7P5** | | | **Autoimmune thrombocytopenic purpura** | | | **Chronic**  **2.8 g/L during labor** | | | **38** | | | **Alive** | | **NA** | | **FAS dysmorphism** | | | **435 g (10th–25th)** | | | **Premature involution** | | | |  | | **Morphometry**  **IHC** | | |  |
| **Case**  **number** | | **Maternal age** | **Gravidity/parity** | | | **Medical history** | | | **Alcohol consumption and others** | | **Term, WG** | | | **IUFD, TOP, alive** | | | **Fetal or neonatal weight (%ile)** | | **General autopsy findings** | | | **Placental weight**  **(%ile)** | | | **Placental lesions** | | | **Brain weight and lesions**  **(%ile)** | | | **Technical investigations** | | |  | |
|  |  | | |  | | |  | | |  | | |  | | |  | |  | |  | | |  | | |  | | | |  | |  | | |  |
| **37** | **40** | | | **NA** | | | **Hepatitis C Gestational diabetes** | | | **Chronic 40 g/day Tobacco** | | | **39** | | | **Alive** | | **3090 g (50th)** | | **FAS dysmorphism** | | | **585 g (75th)** | | | **No lesion** | | | |  | | **Morphometry**  **IHC** | | |  |
| **38** | **43** | | | **G6P5** | | | **Cesarian section for fetal distress IUGR** | | | **Suspected chronic**  **High γGT** | | | **39** | | | **Alive** | | **1630 g (<3rd)** | |  | | | **345 g (<3rd)** | | | **Chorioamnionitis** | | | |  | | **Morphometry**  **IHC** | | |  |
| **39** | **38** | | | **NA** | | | **IUGR** | | | **Chronic not quantified** | | | **40** | | | **Alive** | | **NA** | | **FAS dysmorphism** | | | **344 g (<3rd)** | | | **Premature involution** | | | |  | | **IHC WB** | | |  |
| **40** | **32** | | | **G4P2** | | | **Psychotic disorder**  **Gestational diabetes** | | | **Chronic 50 g/day** | | | **40** | | | **Alive** | | **NA** | |  | | | **600 g (75th)** | | | **No lesion** | | | |  | | **Morphometry**  **IHC** | | |  |
| **41** | **29** | | | **NA** | | | **Alcoholic intoxication withdrawal** | | | **Alcohol suspected**  **Other drugs addiction**  **Increased MGV** | | | **41** | | | **Alive** | | **2640 g (25th)** | |  | | | **385 g (<3rd)** | | | **Excess of fibrin deposits**  **Chronic hypoxia** | | | |  | | **Morphometry**  **IHC** | | |  |
| **42** | **39** | | | **G4P3** | | |  | | | **Chronic 30 g/day** | | | **42** | | | **Alive** | | **2180 g (<3rd)** | |  | | | **430 g (<3rd)** | | | **Excess of fibrin deposits**  **Chronic hypoxia** | | | |  | | **Morphometry**  **IHC** | | |  |

Fetal biometry according to Guihard-Costa et al (2002 ) [17] and Pinar et al (1996) [35]. WG, weeks of gestation; FAS, fetal alcohol syndrome; IHC, immunohistochemistry; IUGR, intrauterine growth retardation; WB, Western blot; TOP, medical termination of the pregnancy; NA, not available; IUFD, intrauterine fetal death.
