# Supplementary material for: PLGF, a placental marker of fetal brain defects after in utero alcohol exposure
Source: Acta Neuropathol Commun. 2017 Jun 6;5:44. doi: 10.1186/s40478-017-0444-6 (PMC5461764; doi:10.1186/s40478-017-0444-6)
Supplement: Supplementary file 6 — Immunohistochemical characteristics of members of the VEGF-PLGF family in human placentae from the “Control” and “Alcohol” groups. (DOCX 25 kb) [file 40478_2017_444_MOESM6_ESM.docx]

**Table S6** Immunohistochemical characteristics of members of the VEGF-PLGF family in human placentae from the “Control” and “Alcohol” groups

| **Control group**  **[20-25 WG[**  **Placental components** | **VEGF VEGF-R1 VEGF-R2 PLGF** | | | |
| --- | --- | --- | --- | --- |
| **Extravillous trophoblasts** | **4+++ ; 1++ ; 2UD** | **2++ ; 5UD** | **2++ ; 5UD** | **1++ ; 2+ ; 4UD** |
| **Decidua** | **5+++ ; 1++ ; 1UD** | **5++ ; 2+** | **1+++ ; 4+ ; 2UD** | **5++ ; 2UD** |
| **Villous trophoblasts** | **6+++ ; 1+** | **6++ ; 1+** | **2+++ ; 4++ ; 1+** | **2++ ; 5+** |
| **Villous vascular endothelial cells** | **3+++ ; 3++ ; 1+** | **1++ ; 6+** | **4+++ ; 2++ ; 1+** | **7+** |
| **Villous vascular smooth cells** | **1+ ; 6UD** | **1+ ; 6UD** | **7+** | **7UD** |
| **Amnios** | **3++ ; 4UD** | **1++ ; 5+ ; 1UD** | **3+++ ; 3++ ; 1+** | **4++ ; 2+ ; 1UD** |
| **Hofbauer cells** | **5+ ; 2UD** | **2++ ; 5+** | **2++ ; 5+** | **4++ ; 3+** |
| **Villous mesenchyma** | **4+ ; 3UD** | **7UD** | **2+++ ; 3++ ; 2+** | **7UD** |
|  |  | | | |
| **Alcohol group**  **[20-25 WG[**  **Placental components** | **VEGF VEGF-R1 VEGF-R2 PLGF** | | | |
| **Extravillous trophoblasts** | **6UD** | **1+++ ; 1++ ; 4UD** | **6UD** | **6UD** |
| **Decidua** | **4+++ ; 1++ ; 1+** | **1+++ ; 2++ ; 2+ ; 1UD** | **1+++ ; 3++ ; 1+ ; 1UD** | **5+ ; 1UD** |
| **Villous trophoblasts** | **5+++ ; 1++** | **3++ ; 2+ ; 1UD** | **3+++ ; 2++ ; 1+** | **1++ ; 4+ ; 1UD** |
| **Villous vascular endothelial cells** | **4+++ ; 1++ ; 1+** | **3+ ; 2-; 1UD** | **3+++ ; 2++ ; 1+** | **5+ ; 1UD** |
| **Villous vascular smooth cells** | **3++ ; 3+** | **6UD** | **2++ ; 4+** | **6UD** |
| **Amnios** | **1++++ ; 1+++ ; 1++ ; 3UD** | **2+; 4UD** | **2+++ ; 1++ ; 3UD** | **4+ ; 2UD** |
| **Hofbauer cells** | **4++ ; 2+** | **1++ ; 1+ ; 4UD** | **1+++ ; 4++ ; 1+** | **4+ ; 2UD** |
| **Villous mesenchyma** | **3++ ; 2+ ; 1UD** | **6UD** | **3++ ; 3+** | **6UD** |

**Table S6 (continued)**

| **Control group**  **[25-35 WG[**  **Placental components** | **VEGF VEGF-R1 VEGF-R2 PLGF** | | | |
| --- | --- | --- | --- | --- |
| **Extravillous trophoblasts** | **2+++ ; 15UD** | **17UD** | **6++ ; 1+ ; 10UD** | **3+++ ; 6++ ; 4+ ; 4UD** |
| **Decidua** | **1+++ ; 11++ ; 5+** | **12++ ; 4+ ; 1UD** | **4++ ; 12+ ; 1UD** | **10++ ; 6+ ; 1UD** |
| **Villous trophoblasts** | **3+++ ; 10++ ; 3+ ; 1UD** | **4++ ; 12+ ; 1UD** | **6+++ ; 5++ ; 5+ ; 1UD** | **7++ ; 9+ ; 1UD** |
| **Villous vascular endothelial cells** | **1+++ ; 1++ ; 14+ ; 1UD** | **16+ ; 1UD** | **7+++ ; 4++ ; 5+ ; 1UD** | **14+ ; 3UD** |
| **Villous vascular smooth cells** | **4+ ; 13UD** | **2+ ; 15UD** | **6+ ; 10Some Cells ; 1UD** | **1+ ; 16UD** |
| **Amnios** | **3++ ; 6+ ; 7UD ; 1amnios nodosum** | **5++ ; 1+ ; 10UD ; 1amnios nodosum** | **3+++ ; 2++ ; 5+ ; 6UD ; 1amnios nodosum** | **1++ ; 9+ ; ; 6UD ; 1amnios nodosum** |
| **Hofbauer cells** | **6+ ; 11UD** | **13+ ; 4UD** | **1++ ; 14+ ; 1UD ; 1Some Cells** | **2++ ; 10+ ; 5UD** |
| **Villous mesenchyma** | **2++ ; 13+ ; 2UD** | **5+ ; 12UD** | **2++ ; 12+ ; 1Some Cells ; 2UD** | **1+ ; 16UD** |
|  |  | | | |
| **Alcohol group**  **[25-35 WG[**  **Placental components** | **VEGF VEGF-R1 VEGF-R2 PLGF** | | | |
| **Extravillous trophoblasts** | **4+++ ; 1++ ; 6UD** | **5++ ; 6UD** | **1++ ; 5+ ; 6UD** | **1++ ; 4+ ; 6UD** |
| **Decidua** | **2+++ ; 9++** | **3++ ; 8+** | **2+++ ; 2++ ; 6+ ; 1UD** | **10+ ; 1UD** |
| **Villous trophoblasts** | **10+++ ; 1+** | **3++ ; 7+ ; 1UD** | **2+++ ; 3++ ; 6+** | **1++ ; 5+ ; 5UD** |
| **Villous vascular endothelial cells** | **3+++ ; 4++ ; 4+** | **4+ ; 7UD** | **3+++ ; 3++ ; 5+** | **5+ ; 6UD** |
| **Villous vascular smooth cells** | **1++ ; 8+ ; 2UD** | **1+ ; 10UD** | **4++ ; 7+** | **1+ ; 10UD** |
| **Amnios** | **1+++ ; 5++ ; 3+ ; 2UD** | **1++ ; 4+ ; 6UD** | **1+++ ; 4++ ; 4+ ; 2UD** | **2++ ; 4+ ; 5UD** |
| **Hofbauer cells** | **6++ ; 3+ ; 2UD** | **7+ ; 4UD** | **1+++ ; 4++ ; 6+** | **1++ ; 3+ ; 7UD** |
| **Villous mesenchyma** | **1+++ ; 3++ ; 7+** | **2+ ; 9UD** | **3++ ; 7+; 1UD** | **1+ ; 10UD** |
|  |  | | | |

**Table S6 (continued)**

| **Control group**  **[35-42 WG[**  **Placental components** | **VEGF VEGF-R1 VEGF-R2 PLGF** | | | |
| --- | --- | --- | --- | --- |
| **Extravillous trophoblasts** | **2+++ ; 17UD** | **1+ ; 18UD** | **5++ ; 14UD** | **5+++ ; 4++ ; 10UD** |
| **Decidua** | **1+++ ; 14++ ; 4+** | **2+++ ; 11++ ; 6+** | **13++ ; 5+ ; 1UD** | **1+++ ; 10++ ; 8+** |
| **Villous trophoblasts** | **3+++ ; 11++ ; 5+** | **9++ ; 10+** | **1+++ ; 10++ ; 7+ ; 1UD** | **1+++ ; 9++ ; 9+** |
| **Villous vascular endothelial cells** | **4++ ; 15+** | **19+** | **1+++ ; 5++ ; 12+ ; 1UD** | **1++ ; 12+ ; 6UD** |
| **Villous vascular smooth cells** | **4+ ; 15UD** | **1+ ; 18UD** | **18+ ; 1UD** | **1+ ; 18UD** |
| **Amnios** | **9+ ; 10UD** | **2++ ; 7+ ; 10UD** | **1+++ ; 3++ ; 9+ ; 6UD** | **11+ ; 8UD** |
| **Hofbauer cells** | **4+ ; 15UD** | **12+ ; 7UD** | **1++ ; 16+ ; 2UD** | **15+ ; 4UD** |
| **Villous mesenchyma** | **1++ ; 15+ ; 3UD** | **5+ ; 14UD** | **18+ ; 1UD** | **19UD** |
|  |  | | | |
| **Alcohol exposed group**  **[35-42 WG[**  **Placental components** | **VEGF VEGF-R1 VEGF-R2 PLGF** | | | |
| **Extravillous trophoblasts** | **24UD** | **24UD** | **4+ ; 20UD** | **1++ ; 8+ ; 15UD** |
| **Decidua** | **1+++ ; 8++ ; 15+** | **7++ ; 16+ ; 1UD** | **2++ ; 20+ ; 2UD** | **1++ ; 19+ ; 4UD** |
| **Villous trophoblasts** | **6+++ ; 9++ ; 9+** | **1++ ; 21+ ; 2UD** | **1+++ ; 3++ ; 17+ ; 3UD** | **15+ ; 9UD** |
| **Villous vascular endothelial cells** | **2++ ; 18+ ; 4UD** | **7+ ; 17UD** | **1++ ; 20+ ; 3UD** | **3+ ; 21UD** |
| **Villous vascular smooth cells** | **3+ ; 21UD** | **24UD** | **2++ ; 10+ ; 12UD** | **1+ ; 23UD** |
| **Amnios** | **16+ ; 8UD** | **2++ ; 8+ ; 14UD** | **3++ ; 11+ ; 10UD** | **7+ ; 17UD** |
| **Hofbauer cells** | **6+ ; 18UD** | **5+ ; 19UD** | **1++ ; 17+ ; 6UD** | **17+ ; 7UD** |
| **Villous mesenchyma** | **2++ ; 14+ ; 8UD** | **24UD** | **1++ ; 13+; 10UD** | **1+ ; 23UD** |

The distribution of immunoreactivy was evaluated using the following scale: 0: no immunoreactivity, +: weak immunoreactivity, ++: moderate immunoreactivity, +++: strong immunoreactivity.

For VEGF immunolabeling, immunoreactivity was strongly detected in extravillous trophoblasts whatever the age of gestation in “Control” and “Alcohol” groups. Decidual cells were strongly positive during 21 and 25 WG, and moderately during the second part of the pregnancy in the two groups. Amniotic cells expressed moderately VEGF between 21 and 34 in both groups, and then decreased. VEGF immunoreactivity was also high between 21 and 25WG in villous trophoblasts in both groups. Between 35 and 42WG, VEGF immunolabeling was low in “Alcohol” group compared with controls. VEGF was weakly expressed in Hofbauer cells in both groups, and negative near the term. Villous mesenchyma was moderately positive whatever the term of the pregnancy in both groups. As regards the villous vasculature, endothelial cell VEGF expression varied among individuals during pregnancy and was negative around the term, although higher in the “Alcohol” group between 27 and 34WG. In vascular smooth muscle cells, VEGF expression was higher in “Alcohol” group between 21 and 34 WG when compared with the control group, with almost no expression at term in both groups.
