# Supplementary material for: PLGF, a placental marker of fetal brain defects after in utero alcohol exposure
Source: Acta Neuropathol Commun. 2017 Jun 6;5:44. doi: 10.1186/s40478-017-0444-6 (PMC5461764; doi:10.1186/s40478-017-0444-6)
Supplement: Supplementary file 7 — Statistical analysis. (DOCX 23 kb) [file 40478_2017_444_MOESM7_ESM.docx]

**Table S7** Statistical analysis

| **Experiments** | **Test** | **n**  **independents experiments** | **p value**  ***p<0.05; **p<0.01; ***p<0.001; ****p<0.0001** |
| --- | --- | --- | --- |
| Cortical vessel disorganization  (Fig. 1c) | Chi-square test | n=4  (4 Ctrl and 4 Alcohol pregnant mice)  *Note: 2 cortex slices were quantified per fetus and 5 to 7 fetuses were analyzed by pregnant mice* | p=0.0032** |
| WB performed in mouse brains  (Fig. 1e, g, h) | Unpaired  t-test | n=6  (6 Ctrl and 6 Alcohol pregnant mice)  *Note: 9 fetus brains were analyzed by pregnant mice* | Fig. 1e p=0.0353*  Fig. 1g p=0.0190*  Fig. 1h p=0.0322* |
| Comparison by WB of brain and placental PLGF levels  (Fig. 1j) | Unpaired  t-test | n=6  (6 Ctrl and 6 Alcohol pregnant mice) | Fig. 1j p=0.0005*** |
| Number of protrusions  (Fig. S1i) | Unpaired  t-test | n=3  (3 Ctrl and 3 Alcohol pregnant mice) | Fig. 2i p=0.0113* |
| Length of protrusions  (Fig. S1j) | Unpaired  t-test | n=3  (3 Ctrl and 3 Alcohol pregnant mice) | Fig. 2j p=0.0034** |
| Reichert's membrane thickness  (Fig. S1k) | Unpaired  t-test | n=3  (3 Ctrl and 3 Alcohol pregnant mice)  *Note: 2 placentae were analyzed per pregnant mice* | Fig. 2k p=0.0047** |
| Number round shape giant trophoblasts  (Fig. S1l) | Unpaired  t-test | n=3  (3 Ctrl and 3 Alcohol pregnant mice) | Fig. 2l p<0.0001**** |
| ELISA PLGF mouse placentae  (Fig. 2i) | Unpaired  t-test  Mann Whitney test | 15 Ctrl and 8 Alcohol pups | Fig. 2t p=0.0013** |
| WB performed in mouse placentae  (Fig. 2b, c, d, e) and (Fig. S2d) | Unpaired  t-test | n=6  (6 Ctrl and 6 Alcohol pregnant mice)  *Note: 9 placentae were analyzed by pregnant mice* | Fig. 2j p=0.0174*  Fig. 2k p=0.0478*  Fig. 2l p=0.0367*  Fig. 2m p=0.0255*  Fig. S1D p=0.0037** (ZO-1)  p=0.0125* (MCT-1) |
| Quantification of recombinant hPLGF  in the fetal brain by ELISA (Fig. 3g) | Unpaired  t-test | n=5  (5 Ctrl fetus brains and 5 fetus brains from injected placentae) | Fig. 3g p=0.0101* |
| *In utero* electroporation of placentae  with *PGF* shRNA  (WB studies)  (Fig. 3l, n) | One way ANOVA  Tukey’s post hoc test | n=3  (3 Sh^-^GFP^-^, 3 Sh^-^GFP^+^ and 3 Sh^+^GFP^+^  pregnant mice)  *Note: For each pregnant mice, 3 placentae were injected* | Fig. 3l ANOVA F 6.466 p=0.0318  Tukey’s p=0.0292*  Fig. 3n ANOVA F 4.920 p=0.0544  Tukey’s p=0.0462* |
| *In utero* electroporation of placentae with *PGF* shRNA (brain vasculature analysis) (Fig. 3r) | Chi-square test | n=3  (3 Sh^-^GFP^-^, 3 Sh^-^GFP^+^ and 3 Sh^+^GFP^+^  pregnant mice)  *Note: For each pregnant mice, 3 placentae were injected* | Fig. 3r p=0.0190*  Ctrl *vs* sh^+^GFP^+^ |
| *In utero* electroporation of placentae with Ctrl CRISPR and *PGF* CRISPR-dCas9 activation plasmids  (Abdomen size) (Fig. 4g) | Two way ANOVA  Tukey’s post hoc test | n=3  (3 non transfected, 3 Ctrl CRISPR and 3 *PGF* CRISPR pregnant mice)  *Note: For each pregnant mice, 3 placentae were injected* | Two-way ANOVA  F (10, 36) = 2.615  p=0.168*  Tukey's multiple comparisons test  *NaCl group*  p=0.0171*  p=0.0079**  *Alcohol group*  p=0.0015**  *vs Ctrl NaCl*  p=0.0083^##^  p<0.0001^####^ |
| *In utero* electroporation of placentae with Ctrl CRISPR and *PGF* CRISPR-dCas9 activation plasmids (Whole size)  (Fig. 4h) | Two way ANOVA  Tukey’s post hoc test | n=3  (3 non transfected, 3 Ctrl CRISPR and 3 *PGF* CRISPR pregnant mice)  *Note: For each pregnant mice, 3 placentae were injected* | Two-way ANOVA  F (10, 36) = 2.615  p=0.168*  Tukey's multiple comparisons test  *NaCl group*  p=0.0011**  p=0.0033**  *Alcohol group*  p=0.0016**  p<0.0001****  *vs Ctrl NaCl*  p=0.0003^###^  p<0.0001^####^  p<0.0001^####^ |
| *In utero* electroporation of placentae with Ctrl CRISPR and *PGF* CRISPR-dCas9 activation plasmids (Head size)  (Fig. S3a) | Two way ANOVA  Tukey’s post hoc test | n=3  (3 non transfected, 3 Ctrl CRISPR and 3 *PGF* CRISPR pregnant mice)  *Note: For each pregnant mice, 3 placentae were injected* | Two-way ANOVA  F (10, 36) = 2.615  p=0.168*  Tukey's multiple comparisons test  *vs Ctrl NaCl*  p=0.0025^##^  p=0.0006^###^ |
| *In utero* electroporation of placentae with Ctrl CRISPR and *PGF* CRISPR-dCas9 activation plasmids (Body size)  (Fig. S3b) | Two way ANOVA  Tukey’s post hoc test | n=3  (3 non transfected, 3 Ctrl CRISPR and 3 *PGF* CRISPR pregnant mice)  *Note: For each pregnant mice, 3 placentae were injected* | Two-way ANOVA  F (10, 36) = 2.615  p=0.168*  Tukey's multiple comparisons test  *Alcohol group*  p=0.0391*  p=0.0032**  *vs Ctrl NaCl*  p=0.0002^###^  p<0.0001^####^  p<0.0001^####^ |
| *In utero* electroporation of placentae with Ctrl CRISPR and *PGF* CRISPR-dCas9 activation plasmids (brain vasculature) (Fig. 4l) | Two way ANOVA  Tukey’s post hoc test | n=3  (3 non transfected, 3 Ctrl CRISPR and 3 *PGF* CRISPR pregnant mice)  *Note: For each pregnant mice, 3 placentae were injected* | Two-way ANOVA  F (2, 12) = 3.806  p=0.0525  Tukey's multiple comparisons test  *Alcohol group*  p=0.0231*  p=0.0171*  *vs Ctrl NaCl*  p=0.0118^#^  p=0.0087^##^ |
| Luminal vascular area per class of villi in human placentae  (Fig. 5d; Fig. S5e) | Unpaired  t-test | 41 placentae in control group  42 placentae in Alcohol group | Fig. 5d p=0.0238* [0-5,000[  Fig. S4e p=0.0101* [0-5,000[ |
| Time course analysis of villous density in placentae  (Fig. 5e) | One way ANOVA Tukey’s post hoc test | 41 placentae in control group  42 placentae in Alcohol group | ANOVA F 21.70 p<0.0001***  Tukey’s post hoc test  p<0.0001^####^  p<0.0001^####^ |
| Analysis of villous density in human placentae between Ctrl and Alcohol groups  (Fig. 5e) | Unpaired  t-test | 41 placentae in control group  42 placentae in Alcohol group | p<0.0001**** |
| Time course of vessel area in placentae  (Fig. 5f) | One way ANOVA Tukey’s post hoc test | 41 placentae in control group  42 placentae in Alcohol group | ANOVA F 3.432 p<0.0355*  Tukey’s post hoc test  p=0.0424^#^ |
| Analysis of vessel area in human placentae between Ctrl and Alcohol groups  (Fig. 5f) | Unpaired  t-test | 41 placentae in control group  42 placentae in Alcohol group | p=0.0113*  p=0.005** |
| WB studies in human placentae (Fig. 5g, i) | Mann Whitney test | n=6  (3 Ctrl placentae and 3 Alcohol placentae) | Fig. 5g p=0.0151*  Fig. 5i p=0.0317* |
| Distribution of villous sizes in human placentae  (Fig. S4c; Fig. S5c) | Unpaired  t-test | n = 41 placentae in Ctrl group  n= 42 placentae in Alcohol group | Fig. S3c p=0.0303* [0-5,000[  Fig. S4c p=0.0239* [0-5,000[  Fig. S4c p= 0.0129* [5,000-10,000[ |
| Distribution of vessels per class of villi in human placentae  (Fig. S5d) | Unpaired  t-test | 41 placentae in control group  42 placentae in Alcohol group | Fig. S4d p=0.0392* [0-5,000[  Fig. S4d p=0.0166* [5,000-10,000[ |
| Correlation between cortical and placental vascular impairments  (Fig. 6i, j) | Ozone Correlation | Analysis integrating three gestational ages  [20-25WG[, [25-35WG[ and [35-42WG] | *Control group*  r^2^= 0.4719  p (two-tailed)>0,9999  *Alcohol group*  R²= 0.9895  p (two-tailed)=0.3333 |
