# Supplementary figures and images for: PLGF, a placental marker of fetal brain defects after in utero alcohol exposure
Source: Acta Neuropathol Commun. 2017 Jun 6;5:44. doi: 10.1186/s40478-017-0444-6 (PMC5461764; doi:10.1186/s40478-017-0444-6)

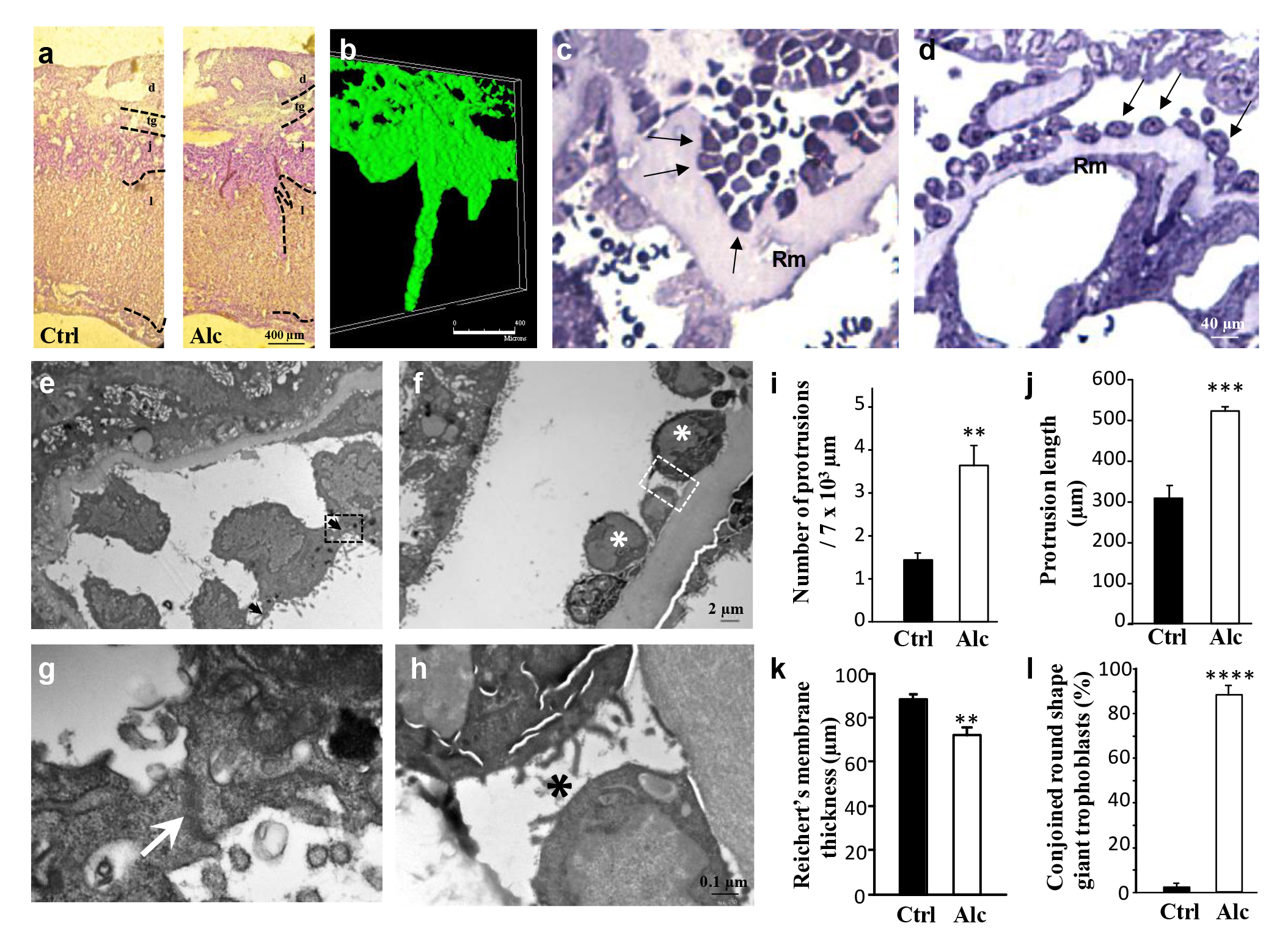

Supplement: Supplementary file 8 — Effects of in utero alcohol exposure on morphometric and ultrastructural characteristics of the placenta from GD20 mice. (a) Visualization by Cresyl violet staining of the effect of alcohol exposure on the laminar structuration of the placenta. The placenta is oriented with its maternal side at the top. Note that alcohol induced protrusions of the junctional zone within the labyrinth zone (dotted lines). (b) Visualization of a typical 3D reconstruction of placental protrusions used for morphometric analysis. (c-d) Visualization at low magnification of the giant trophoblast layer from control (c) and alcohol-exposed (d) groups. Arrows indicate giant trophoblasts. Note the typical rectangular shape of this cell type in placentae from the control group whereas in the alcohol-exposed group the trophoblasts present a round shape. (e-h) Images acquired by electron microscopy at moderate (e and f) and high (g and h) magnifications visualizing the morphology of giant trophoblasts and the presence of zonula occludens (arrows) from control (e and g) and alcohol-exposed (f and h) groups. Note a loss of zonula occludens (stars) in alcohol-treated animals. Inserts present in e and f indicate the area visualized at high magnification in g and h, respectively. d: maternal decidua; j: junctional zone; l: labyrinth zone; tg: trophoblast giant layer. (i-l) Quantification by morphometric analysis of the effect of alcohol on the number of placental protrusions (i), the length of protrusions (j), the thickness of the Reichert’s membrane (k) and the proportion of round-shape giant trophoblasts in control and alcohol-exposed placentae (l). *p < 0.05; **p < 0.01; ****p < 0.0001 vs the control group using the unpaired t test. (TIFF 15056 kb) [file 40478_2017_444_MOESM8_ESM.tif]

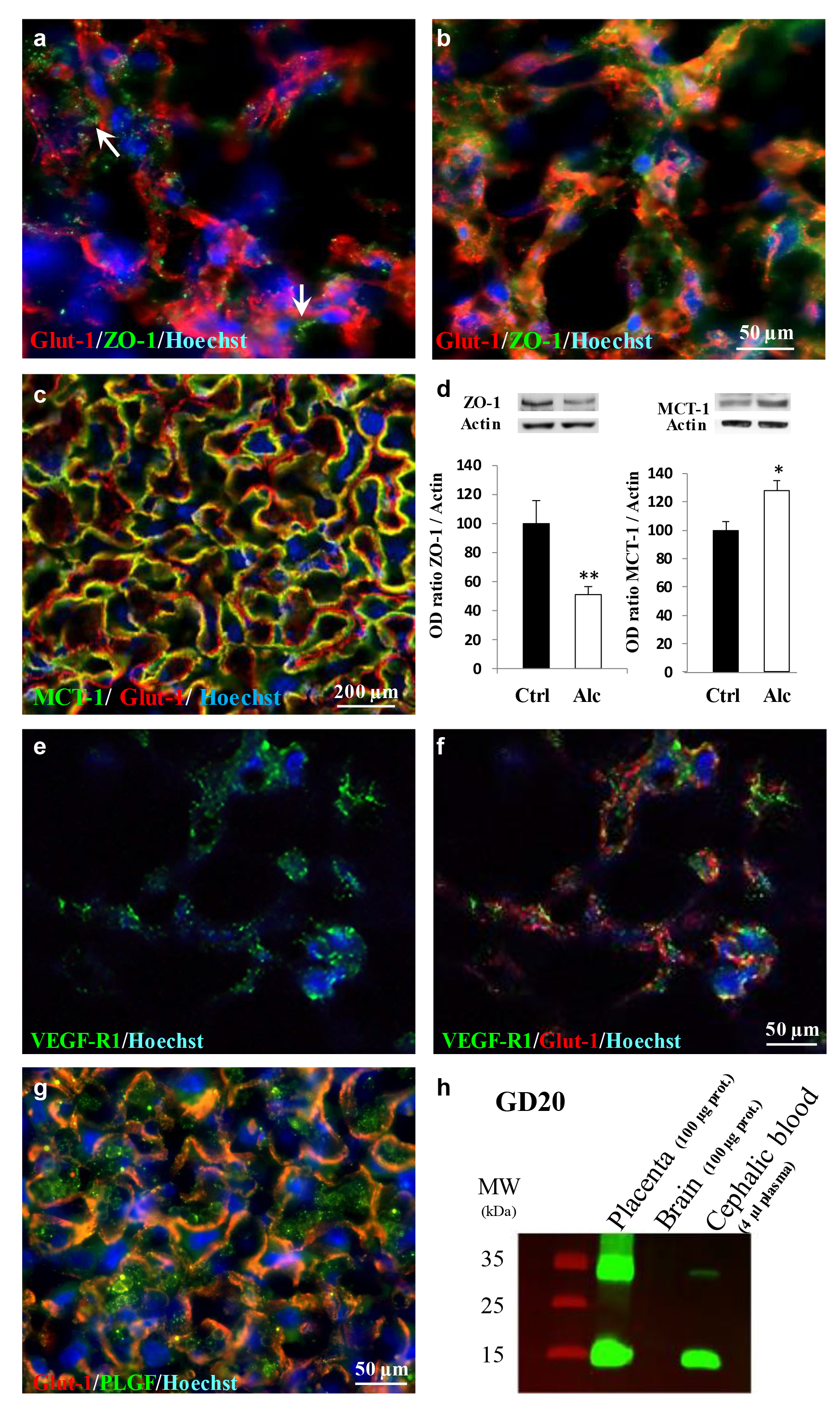

Supplement: Supplementary file 9 — Effects of in utero alcohol exposure on ZO-1 and MCT-1 expression and visualization of VEGF-R1 in the mouse placenta. (a, b) Visualization by immunohistochemistry of the ZO-1 protein in the labyrinth zone of the mouse placenta from the control (a) and the alcohol-exposed (b) groups. Note that ZO-1 immunolabeling is dotted and clustered (arrows) in the control group whereas it is diffuse in the alcohol-exposed group. Immunoreactivity against the glucose transporter Glut-1 was done to visualize the trophoblast layers. Hoechst was used to label nuclei. (c) Double immunolabeling experiment performed with the monocarboxylate and the glucose transporters MCT-1 and Glut-1, respectively in the labyrinth zone of a control placenta. Note that, contrasting with Glut-1, MCT-1 expression is associated with one syncytiotrophoblast layer (maternal side). Hoechst was used to label nuclei. (d) Quantification by Western blot of the expression levels of the proteins ZO-1 and MCT-1 in the placentae from control and alcohol-exposed groups. Western blot experiments showed that placentae from alcohol-exposed animals had significantly decreased ZO-1 levels while MCT-1 protein levels were significantly increased. *p < 0.05, **p < 0.01 vs the control group using the unpaired t test. (e, f) Immunohistochemistry experiments illustrating the distribution of VEGF-R1 (e) and Glut-1 (f) in the syncytiotrophoblast layers of the mouse placenta. Hoechst was used to visualize nuclei. (g) Immunohistochemistry experiments visualizing Glut-1 and PLGF immunoreactivity in the syncytiotrophoblast layers of the mouse placenta. Hoechst was used to visualize nuclei. (h) Visualization by Western blot of PLGF in 100 μg protein extracts from GD20 placenta and E20 brain and in 4 μl plasma from E20 cephalic blood. (TIFF 23009 kb) [file 40478_2017_444_MOESM9_ESM.tif]

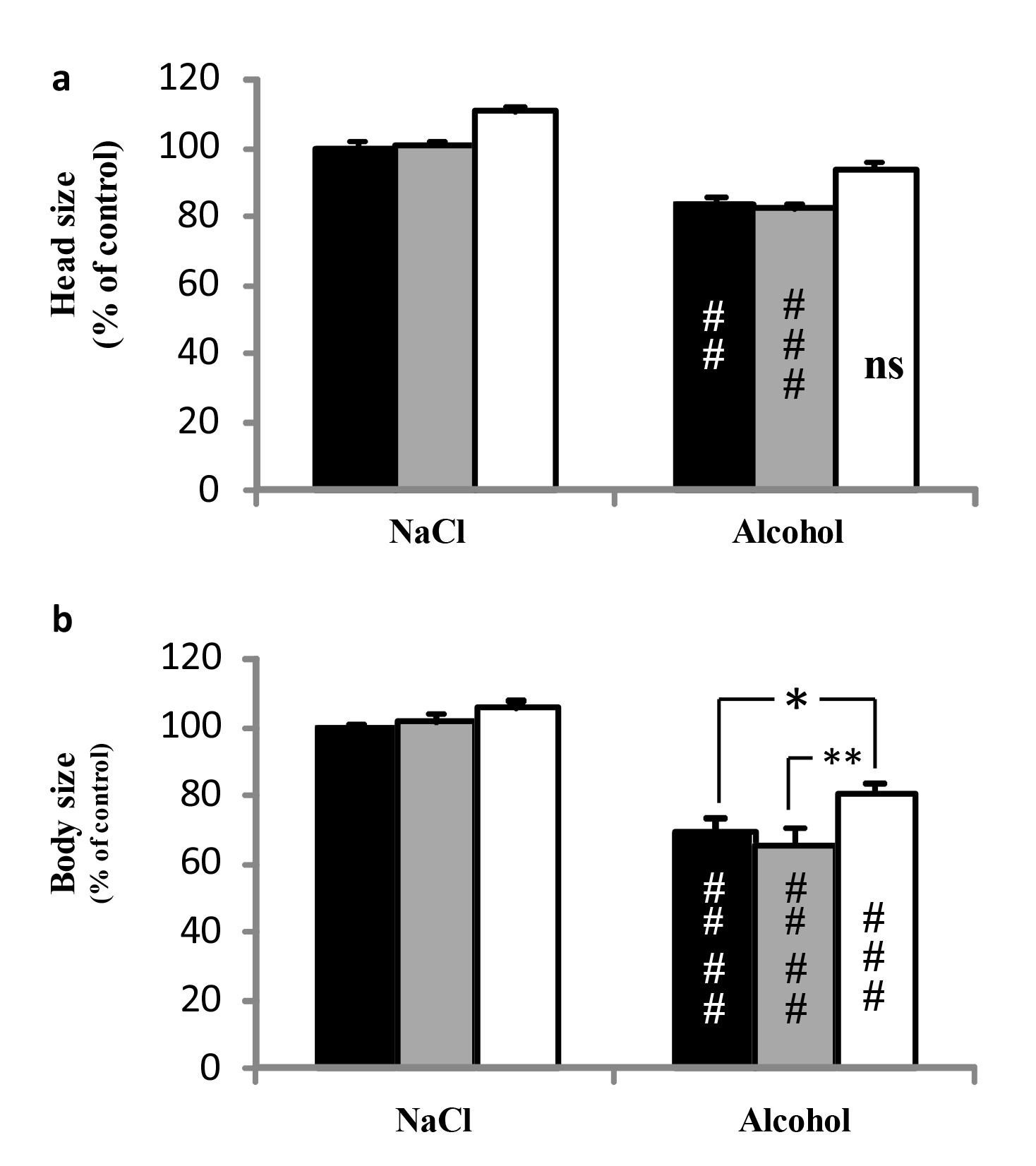

Supplement: Supplementary file 10 — Effects of placental in utero PGF overexpression on head and body sizes of E20 fetuses in control and alcohol groups. (a, b) Quantification of head (a) and body (b) sizes in control (NaCl) et alcohol groups. In a same uterine horn some placentae were not electroporated (black bars), electroporated with control CRISPR-Cas9 plasmids (grey bars) or electroporated with PGF CRISPR-dCas9 plasmids (white bars). ##p < 0.01; ###p < 0.001; ####p < 0.0001 vs the control group and *p < 0.05; **p < 0.01 as indicated using the two way ANOVA test followed by Tukey’s post hoc test. (TIFF 7601 kb) [file 40478_2017_444_MOESM10_ESM.tif]

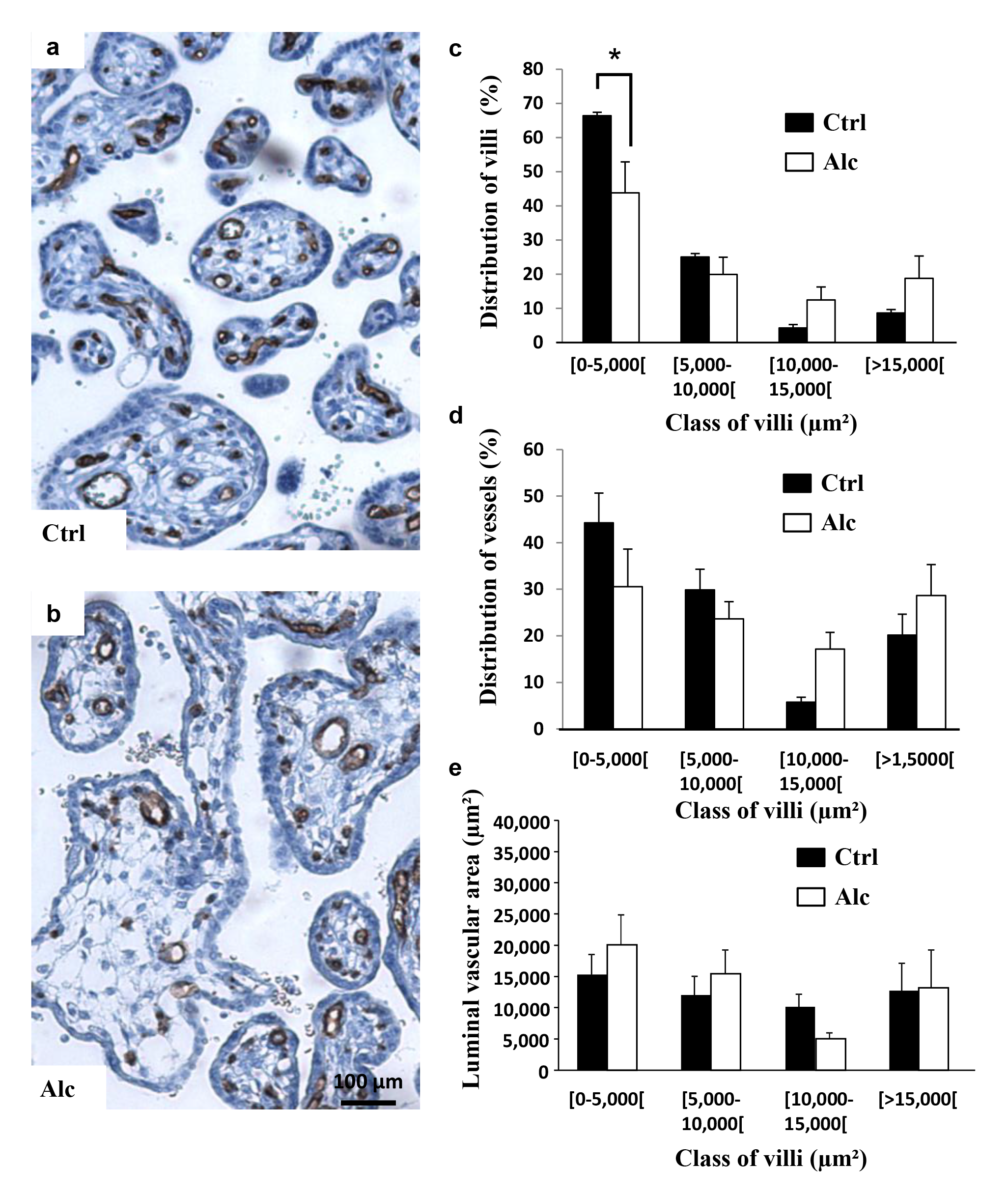

Supplement: Supplementary file 11 — Histomorphometric characterization of the effects of in utero alcohol exposure on human placentae from WG20 to WG25. (a, b) Immunohistochemistry performed against CD31 and toluidine blue counterstaining visualizing microvessels (brown) present in placental villi (blue) from control and alcohol-exposed groups collected at gestational ages ranging from [20–25 WG]. (c) Percentage of villi classified by sizes in placentae from control and alcohol-exposed groups collected at gestational ages ranging from [20–25 WG].*p < 0.05 vs the control group using the unpaired t test (d) Repartition of vessels per size of villi in placentae from control and alcohol-exposed groups collected at gestational ages ranging from [20–25 WG]. (e) Luminal vascular area per size of villi in placentae from control and alcohol-exposed groups collected at gestational ages ranging from [20–25 WG]. (TIFF 19794 kb) [file 40478_2017_444_MOESM11_ESM.tif]

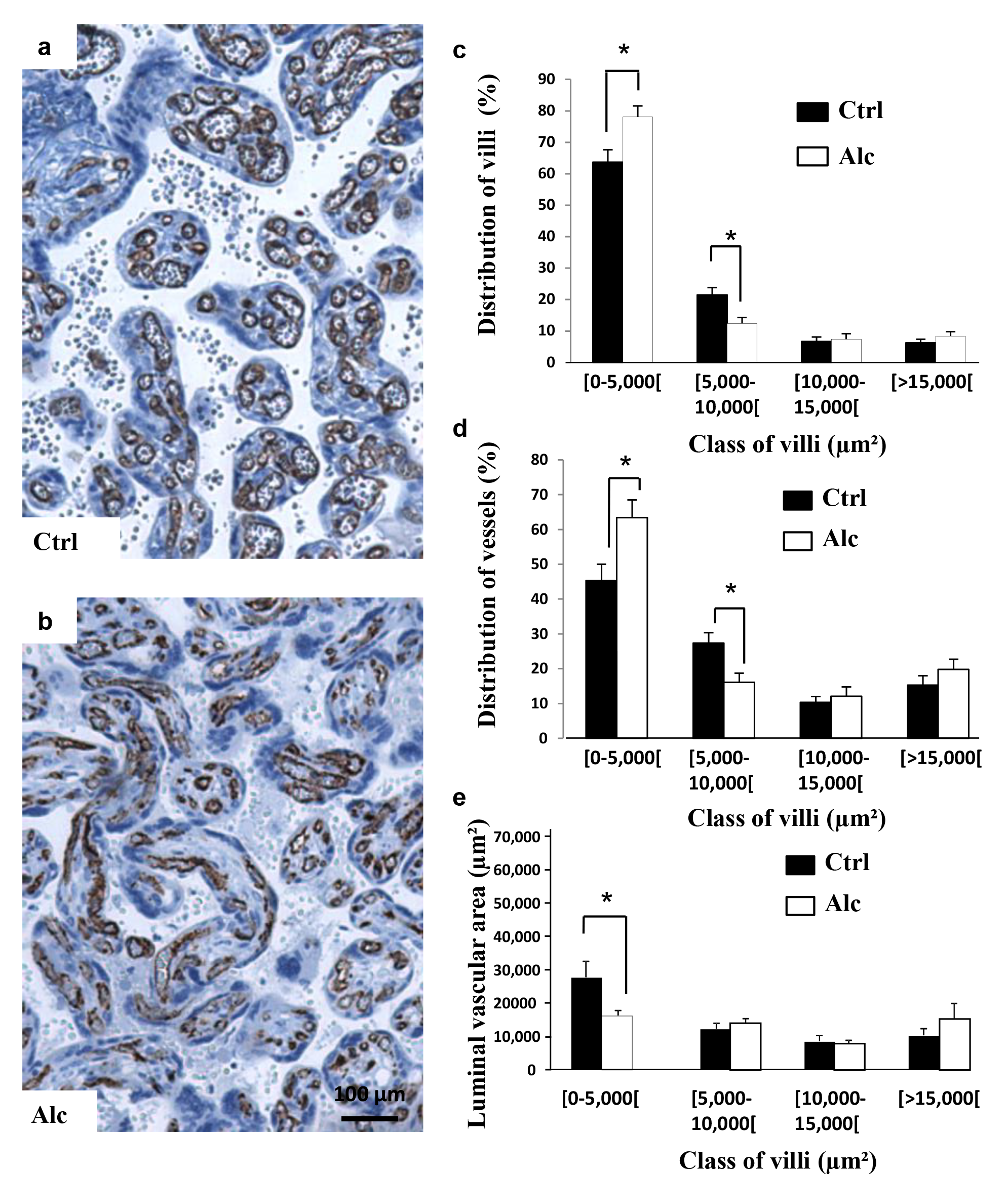

Supplement: Supplementary file 12 — Histomorphometric characterization of the effects of in utero alcohol exposure on human placentae from WG25 to WG35. (a, b) Immunohistochemistry performed against CD31 and toluidine blue counterstaining visualizing microvessels (brown) present in placental villi (blue) from control and alcohol-exposed groups collected at gestational ages ranging from [25–35 WG]. (c) Percentage of villi classified by sizes in placentae from control and alcohol-exposed groups collected at gestational ages ranging from [25–35 WG]. *p < 0.05 vs the control group using the unpaired t test. (d) Repartition of vessels per size of villi in placentae from control and alcohol-exposed groups collected at gestational ages ranging from [25–35 WG]. *p < 0.05 vs the control group using the unpaired t test. (e) Luminal vascular area per size of villi in placentae from control and alcohol-exposed groups collected at gestational ages ranging from [25–35 WG]. *p < 0.05 vs the control group using the unpaired t test. (TIFF 19271 kb) [file 40478_2017_444_MOESM12_ESM.tif]

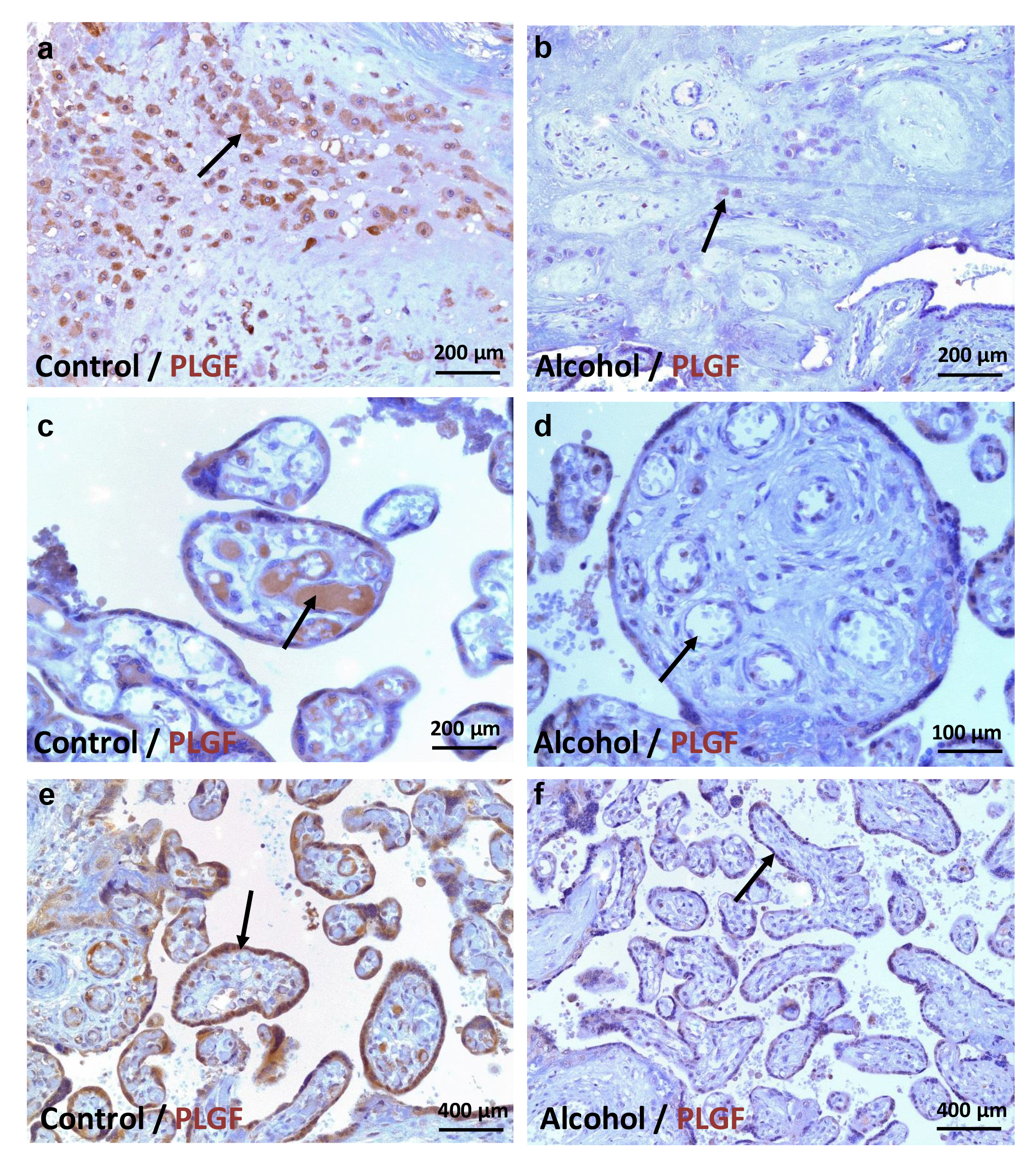

Supplement: Supplementary file 13 — PlGF immunoreactivity of the main placental compartments in control and prenatally alcohol-exposed neonates for gestational ages [35–42 WG]. (a, b) Strongly immunoreactive decidual cells in a control placental maternal floor (arrow), conversely to those of a prenatally alcohol-exposed neonate, where decidual cells exhibit a weak immunoreactivity (arrow). (c, d) Circulating PlGF in the villous capillaries (arrow) in a normal placenta at term, contrasting with absent intra-luminal PlGF immunoreactivity in the villous vessels of a prenatally ethanol-exposed neonate (arrow). (e, f) Strong PlGF immunoreactivity of the villous syncytiotrophoblasts in a control placenta at term (arrow), contrasting with weak and irregular PlGF immunoreactivity in the villous trophoblasts in a prenatally ethanol-exposed neonate (arrow). (TIFF 25948 kb) [file 40478_2017_444_MOESM13_ESM.tif]
